# Cybersecurity vulnerabilities of variable speed limit signs and their effects on accident risk and traffic safety

**DOI:** 10.1038/s41598-026-51168-9

**Published:** 2026-05-06

**Authors:** Maryam Samaei, Mostafa Ameli, Samaneh Yazdanipour, Reza Arani

**Affiliations:** 1 GRETTIA, COSYS, Univ. Gustave Eiffel, Paris, France; 2 Department of Electrical Engineering and Computer Sciences, University of California, Berkeley, Berkeley, USA; 3 Department of Electrical, Computer and Biomedical Engineering, Toronto Metropolitan University, Toronto, M5B 2K3 ON Canada

**Keywords:** Variable speed limit (VSL), Disruption, SUMO, Safety, Threat model, Engineering, Civil engineering

## Abstract

Variable Speed Limits (VSL) are primarily designed to enhance traffic safety on hazardous roadways by dynamically adjusting speed limits based on real-time traffic and weather conditions. Most previous studies have focused on using VSLs to improve safety, mitigate congestion, and reduce environmental impact, assuming a securely connected VSL network. But what would be the impact of cyberattacks on VSL systems on traffic safety and network efficiency. This paper investigates the impact of intentional disruptions on VSL signs within traffic networks. We present a threat model to identify vulnerabilities in the VSL communication network and potential access points for attackers. An analytical accident model was developed, and various VSL disruption scenarios were simulated using a case study of Highway 1 in British Columbia, Canada, in SUMO (Simulation of Urban MObility) to assess the potential for crashes and safety problems resulting from VSL disruptions. Surrogate Safety Measures (SSM) were employed as key measures of safety concerns. Our findings highlight notable risks, including up to 56$\%$ additional conflicts, posed by these intentional disruptions. A robustness assessment using Monte Carlo simulation runs of a scenario with heterogeneous driver behaviors demonstrated that the safety impact is consistent and not random seed-dependent. Across runs, intentional disruptions still yielded significant increases in conflicts, with an average rise of 24.95%, confirming the stability of the results. Various mitigation strategies are discussed to enhance traffic safety and resilience against VSL manipulation.

## Introduction

Disruptions in transportation networks can have significant impacts on traffic flow, safety, and overall network efficiency and cause Direct and indirect cost for system and end-users. These disruptions may arise from natural events or human actions. While natural disasters have been extensively studied in the literature^1^, human-induced disruptions have not received sufficient attention, despite their potential to cause more severe damage and incur higher costs, as they can be intentional and strategically targeted.

With the increasing deployment of Intelligent Transportation Systems (ITS), the cyber-physical security of transportation is becoming a more critical issue for traffic control and management. Over the last decade, numerous intentional transportation disruptions have occurred due to cyber intrusion. For instance, in 2020, an artist tricked Google Maps by creating a virtual traffic jam in Berlin, impacting real-world traffic by rerouting vehicles to avoid the nonexistent congestion^2^. In August 2017, a variable message sign in California was hacked, displaying offensive messages. This incident resulted in distractions and safety concerns for drivers^3^. In 2016, a man manipulated a variable message sign to display a humorous message, which could potentially distract drivers and raise safety concerns. More examples of transportation disruptions can be found in relevant literature^4^. Existing transportation research often focuses on vulnerability assessments of transportation networks against natural hazards or traffic signal disruptions, with fewer studies examining targeted disruptions to other ITS infrastructures. However, new technologies, such as Variable Speed Limit (VSL) signs, are rapidly emerging and may pose significant safety threats if not properly secured and monitored.

VSLs are used to dynamically control traffic by adjusting speed limits based on real-time data and weather conditions. They are prevalently used in areas with adverse weather conditions and in some locations to improve safety and traffic conditions using different control strategies^5^. However, they are vulnerable to cyber-physical attacks and can be the source of safety issues. In the literature, variable speed limits (VSLs) have been studied and applied for multiple purposes, including enhancing safety on hazardous road segments^6^, reducing the environmental impacts of traffic^7^, and mitigating congestion through optimized control strategies^8,9^. Despite these efforts, the resilience of VSL systems to cyber intrusions and the consequences of their disruption on traffic safety and operational conditions have received little attention. The objective of this paper is to highlight the potential disruptions caused by the manipulation of VSLs and to assess the associated impacts. Additionally, with the emergence of driverless vehicles and the increasing use of AI, the cybersecurity of transportation infrastructure has become more crucial than ever. In fact, we seek to evaluate the vulnerability of VSLs, which are primarily designed to improve safety but, if tampered with, could themselves become a source of safety risks. To the best of our knowledge, no study has yet investigated the vulnerability of VSLs to such intrusions. To address this research gap, this study makes the following contributions:

- The susceptibility of VSL systems to cyber-physical intrusions is analysed for the first time using a threat-modelling approach.
- An analytical model is developed to assess the likelihood of collision or near-collision scenarios resulting from malicious VSL manipulation.
- The effects of VSL disruptions on overall network safety and traffic dynamics are evaluated through a simulation case study.
- Potential countermeasures to prevent or reduce the impact of cyber-physical attacks on VSLs are proposed and discussed.

The subsequent sections of this paper will provide a literature review on VSLs and cybersecurity within the transportation sector (Related works section). Next, we describe the methodological approach used to investigate the vulnerabilities of VSLs and the effects of cyber-physical attacks on them on traffic safety and network efficiency (Methodology section). Then we present a common communication framework and a review of the vulnerabilities associated with VSLs (Threat model section). An analytical model representing the crash risk caused by VSL manipulation will then be detailed (Theoretical analysis section). We then assess various disruption scenarios using a simulation case study of Highway 1 in British Columbia, Canada, to identify critical times and locations in a real-world context (Problem overview and simulation results section). The results are analyzed, and potential mitigation strategies are proposed (Discussion section). Finally, the paper will conclude with recommendations for future research (Conclusion section).

## Related works

VSLs are among the most effective traffic management methods, proven to harmonize speeds and reduce travel times^10^. They can also enhance safety when appropriate speed limits are set based on real-time traffic conditions, by reducing the frequency of short headways and decreasing speed differences^11^. The related literature proposes various control algorithms for VSLs. For instance^12^, employed a multi-agent Reinforcement Learning (RL) algorithm to alleviate congestion at consecutive freeway bottlenecks by enhancing speed homogeneity across the entire freeway^13^. developed safety performance functions for VSL/Variable Advisory Speed (VAS) using microscopic traffic detectors and detailed VSL/VAS operation data to investigate crash frequency and its associated parameters. Additionally^14^, proposed a VSL algorithm in cooperation with autonomous vehicles to reduce stop-and-go waves at traffic signals and reduce energy consumption.

In general, VSLs have been studied with three primary objectives: congestion mitigation^12,15–17^, safety improvement^6,13,18^, and environmental benefits^7,14^. It is noteworthy that some above studies focus on multiple objectives simultaneously, and all reported improvements in traffic flow, safety, and environmental outcomes. There is also a study that examined the temporal and localized effects of VSLs on traffic safety, specifically focusing on aspects such as speed reduction behavior and the impact of social pressure from surrounding traffic^19^. However, all above studies assume a connected infrastructure without any intentional disruption. Despite their benefits, these infrastructures are vulnerable to cyberattacks, which can adversely affect safety and traffic management.

Vulnerability and resilience of transportation systems against cyber-physical disruptions are critical issues due to their potential to impact large populations and other interconnected sectors. The literature has extensively examined traffic network vulnerability and resilience assessments, focusing on identifying key nodes and edges using graph theory^20–22^.

With the rising frequency of disruptions in recent years, and the increasing prevalence of ITS, several studies have specifically addressed network vulnerability to targeted disruptions. Some studies have explored the impact of disinformation attacks on transportation networks^23^. examined the effects of misleading drivers either towards or away from certain locations on additional travel time. The study further identified areas that would maximize disruption cost by solving an optimization problem.

Many researchers have focused on the vulnerability of traffic signals and their effects on traffic networks. For instance^24^, examined the vulnerability of the network to disruptions in traffic signals, identifying traffic lights that impact traffic congestion and travel times significantly^25^. proposed an algorithm based on game theory to detect and mitigate the tampering of traffic signals. They validated their algorithm through numerical simulation experiments conducted using the traffic simulator Simulation of Urban MObility (SUMO)^26^. explored various types of attacks on traffic signal infrastructure, and^27^investigated the vulnerability of electric vehicle fleets in car-sharing services by analyzing changes in route distance, passenger delivery times, and vehicle charging costs, assuming disruptions caused by manipulated traffic lights and sensor data^28^. addressed the cybersecurity challenges faced by urban traffic control systems and proposes resilient perimeter control algorithms designed to enhance the control system vulnerabilities.

While numerous studies have examined the manipulation of traffic signals, detectors, and autonomous vehicles, see, e.g^29–31^.,, limited studies have explored the vulnerabilities of other ITS infrastructures. For example^32^, investigated various attack methods on variable message signs and discusses potential mitigation strategies. Similarly^33^, evaluated the impact of attacks on connected environments, such as Vehicle-to-Everything (V2X) advisory speed limits, analyzing transportation indicators such as average waiting time and network flow through simulations. However, to the best of our knowledge, no study has specifically investigated cyberattack against VSLs. In our study, we examine the cyber-physical network associated with VSLs and identify potential vulnerabilities that could be exploited by adversaries.

## Methodology

**Fig. 1:**
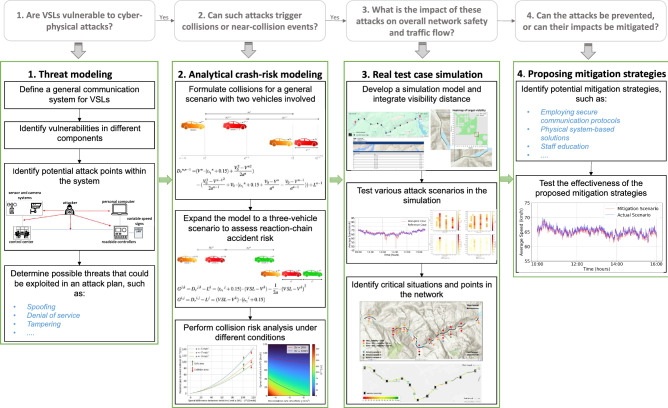
Overview of the methodological framework used in this study. The top row presents the key research questions, and the bottom row shows the corresponding methods used to address each question.

In this section, the methodological process of the study is presented. Figure 1 illustrates the overall workflow. The upper row summarizes the key research questions, while the lower row outlines the methods used to address each question. This section provides a brief overview of the methods for each step. Detailed explanations can be found in the respective sections.

In the first step, we investigate whether VSL systems are vulnerable to cyber-physical attacks. To answer this question, we conduct an analysis to identify vulnerabilities within the VSL communication architecture. After establishing that VSLs are indeed susceptible to intrusions, the second step focuses on determining whether such attacks can lead to safety issues, including collisions or near-collision situations. This question is examined through an analytical crash-risk model, which demonstrates that malicious VSL manipulation can result in collisions under certain conditions. The third step assesses the broader impact of these attacks on network-level safety and traffic flow. To do so, we use a real test-case simulation that incorporates VSL operations and models multiple intrusion scenarios. This allows us to quantify the system-wide effects of VSL disruptions and to identify critical locations or conditions where an attack would have the most severe consequences. Finally, we address the question of how such attacks can be prevented or mitigated. We propose several mitigation strategies and evaluate their effectiveness within the simulation framework. Overall, the methodological framework presented in Figure 1 is generic and can be applied to other similar cyber-physical risk assessment studies involving traffic control systems.

## Threat model

The ultimate goal of the attacker is to create disruptions in the transportation system by manipulating VSL sign display. Manipulated VSLs can mislead drivers into making wrong decisions, leading to congestion or accidents on the road. This section introduces the threat model of these attacks. Threat modeling is used to identify vulnerabilities within a system^34^. In this section, we identify the components of a VSL sign cyber-physical network, explore the vulnerabilities that attackers can exploit, and discuss the feasibility of these vulnerabilities being used for attacks. The possible physical impacts and mitigation solutions are discussed in later sections.

**Fig. 2:**
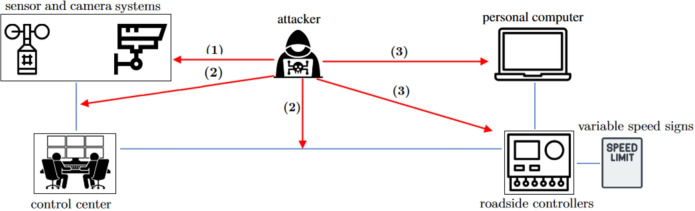
Communication network of Variable Speed Limit (VSL) system. Blue arrows represent communication flows between components, whereas red arrows highlight potential intrusion points that attackers could exploit to carry out cyber-physical attacks.

Figure 2 illustrates a common cyber-physical network used for the implementation of VSLs. This network comprises four fundamental components: sensor and camera systems, a control center, roadside controllers, and digital variable speed signs. Online data on weather and traffic conditions, collected from multiple sensors, including those for weather, traffic, pavement, and visibility, are essential inputs to calculate the appropriate speed limit^35^. These inputs are subsequently transmitted to the control center, where algorithms process the data to determine appropriate speed limits. The calculated speed limits can be reviewed and revised by human experts in the control center if necessary. The finalized speed limits are then sent to roadside controllers, enabling dynamic adjustment of speed limits as needed.

As VSLs are typically deployed on dangerous roads to provide real-time, appropriate driving speeds, it is crucial to recognize potential vulnerabilities within the system, as adversaries could exploit various access points. In Figure 2, communications between different components are depicted with blue lines, the attacker is illustrated at the center, and potential access points to the system are represented with red arrows. These potential access points are as follows:

- **Direct sensor or camera access (access point 1)**: Adversaries may directly manipulate sensors or cameras, as physical access to these devices is relatively feasible since they are located on the road. However, manipulating sensor data presents a considerable challenge, as attackers must alter input parameters in a manner that yields their desired outputs from the internal algorithms. For an effective attack, the attacker needs some knowledge of the internal algorithms, and each algorithm requires a specific study.
- **Communication between sensors and control center, or between the control center and roadside controller (access points 2)**: Attackers may manipulate communications among different parts of the system to disrupt its functionality. These communications, whether wired or wireless, are typically kept private due to security concerns. However, interrupting wireless communications is generally easier than their wired equivalents. Given that variable speed signs are predominantly deployed along highways, covering extensive stretches of road, wireless communication is favored and consequently more susceptible to attacks. This susceptibility increases when utilizing Simple Network Management Protocol (SNMP) V1 or V2^36^ communication protocols, which lack encryption and authentication features, particularly common in some areas of North America. Updating these protocols entails not only software upgrades but also hardware modifications, complicating the process. Nevertheless, in this study, it is hypothesized that the communication protocol employed in variable speed signs is SNMPv3, given their installation date after the year 2015^37^. That ensures both encryption and authentication, which enhances resistance to attacks.
- **Direct roadside controller access (access point 3)**: Attackers can directly manipulate the roadside controller or connect to it using a computer. Physical access to these controllers is not difficult as they are situated along the roadside. System operators who can access these controllers may have different levels of access^38^, with the highest level allowing them to override commands sent by the control center and manually change the speed limit. Thus, the attacker in this scenario could be an insider or someone with the authentication credentials of a system operator.

Among all potential access points discussed, accessing and controlling sensors and cameras, or interfering with communication between sensors and the control center, as well as between the control center and roadside controllers, seem more challenging compared to the direct manipulation of roadside controllers. Consequently, this study focuses on scenarios where attackers exploit direct access to roadside controllers for potential attacks. In such cases, attackers may employ different actions to put the system under threat.

In general, six categories of threats are proposed by Microsoft: Spoofing, Tampering, Repudiation, Information Disclosure, Denial of Service, and Elevation of Privileges^34^. In our study, the attacker needs to gain access to the roadside controller. Such attacks might be carried out by an insider or someone with stolen credentials. The attacker can achieve this access through a series of initial attacks, including spoofing, where an attacker impersonates a legitimate user to gain access to the system, and/or elevation of privileges, where the attacker acquires unauthorized access to the system. Eventually, after obtaining the necessary access, the attacker needs to conduct a denial of service attack, where they prevent the control center from updating the speed limits, or tampering, where the attacker alters the speed limit to be displayed.

Based on the discussion above, cyber intrusions are feasible, allowing adversaries to potentially manipulate speed limits at any time and location. In the following sections, we will first examine the potential for car accidents resulting from VSL manipulation and then present simulated attack scenarios.

## Theoretical analysis

In this section, we analyze the potential for vehicle accidents resulting from VSL manipulation. Given that our case study primarily involves a single-lane scenario, we will use an analytical model specifically designed for rear-end collisions. Additionally, we extend our model to assess the risk of chain-reaction accidents in car-following scenarios. Table 1 presents the notations used in this analysis, along with their descriptions.

To determine the likelihood of a collision in a car-following scenario, it is essential to explore the feasibility of maintaining the required distance between vehicles. The safety distance usually represents the distance required between a follower and a leader when the leader breaks and stops suddenly. In other words, the safety distance is generally the distance required to prevent collision with a stopped leader or obstacle. However, in many situations, vehicles do not need to stop completely; instead, they should adjust their speed to follow the leader vehicle when it slows down. In such cases, if the distance between vehicles is insufficient when breaking is necessary, it indicates an increased likelihood of a collision. Here, we first calculate and discuss the minimum required safety distance between vehicles in a two-vehicle scenario, and then extend the analysis to a three-vehicle scenario considering a false VSL sign; this means both follower and leader vehicles follow VSL speed while the leader breaks to achieve a lower speed after recognizing the VSL error due to the vehicles/congestion in front.

**Fig. 3:**
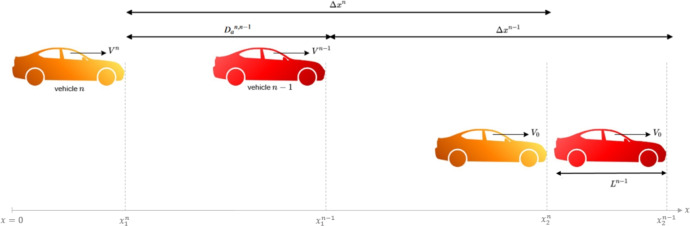
A general car-following scenario illustrating the follower vehicle *n* and its leader $n-1$. The longitudinal baseline is shown by the x-axis, along which the vehicle positions $x^{n}$ and $x^{n-1}$ are defined. The initial spacing between the vehicles $D_{a}^{n,n-1}$, the leader’s length $L^{n-1}$, and the respective displacements $\Delta x^{n}$ and $\Delta x^{n-1}$ are also depicted. These elements are used in deriving the minimum safety distance presented in Equations 1-6.

Figure 3 depicts a general scenario involving two vehicles, a follower vehicle, denoted as Vehicle $n$, and its leader, Vehicle $n-1$. As shown in Fig. 2, the longitudinal baseline is indicated by the x-axis arrow, along which the position of vehicles is defined. $x_1^{n}$ represents the initial position of the follower vehicle and $x_1^{n-1}$ represents the initial position of leader vehicle.

**Fig. 4:**
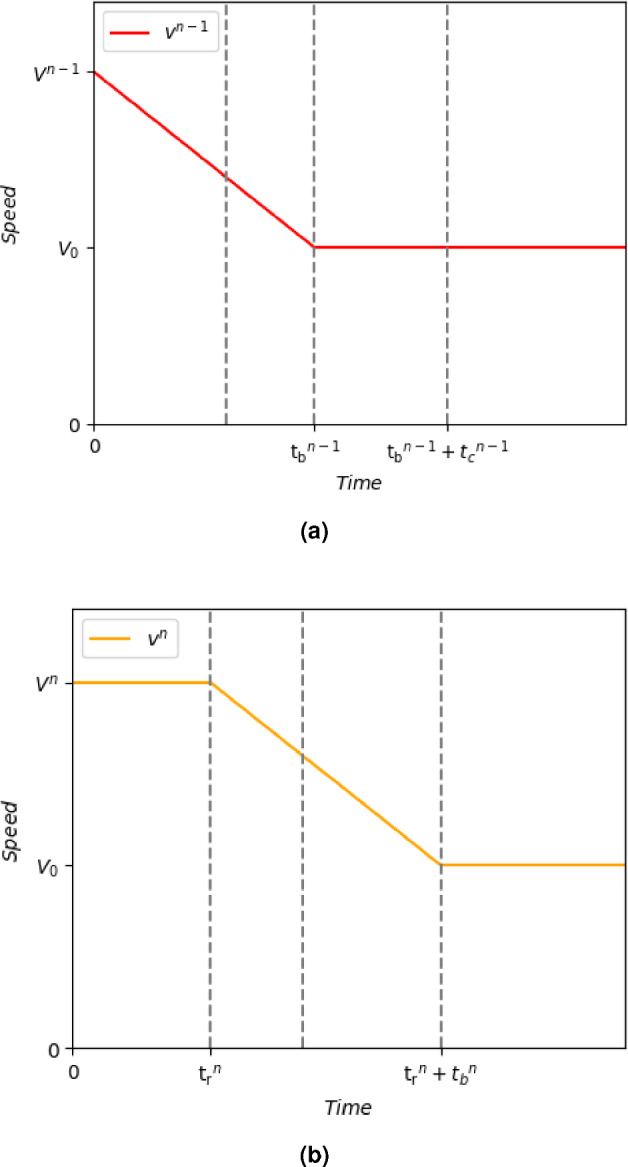
Speed–time diagrams for the leader and follower vehicles in the general scenario. **(a)** The leader starts at speed $V^{n-1}$, brakes uniformly to $V_0$ over $t_b^{n-1}$, and then continues at constant speed. $t_c^{\,n-1}$ denotes the additional cruising duration at the final speed $V_0$. **(b)** The follower traveling initially at $V^n$, reacts after a reaction–reaction time $t_r^{n}$, and brakes to the same final speed $V_0$ over $t_b^{n}$. Both vehicles converge to the same final speed $V_0$ at $t_b^{n-1} + t_c^{\,n-1} = t_r^{n} + t_b^{n}$.

Figure 4 showcases the respective speed-time graphs of the follower and the leader in the general scenario. In this scenario, Vehicle $n-1$ is driving at speed $V^{n-1}$ when it decides to decelerate to reach the speed $V_0$ ($V_0 < V^{n-1}$). Meanwhile, Vehicle $n$ is following Vehicle $n-1$ at speed $V^n$ when Vehicle $n-1$ initiates breaking. Vehicle $n$ must first recognize the leader’s speed reduction and then react by decelerating to match or fall below the new speed of Vehicle $n-1$($V_0$). This time period, called reaction time and denoted by ${t_r}^n$, is indicated in Figure 4.

In Figure 3, $x_2^{n}$ and $x_2^{n-1}$ represent the secondary position of follower and leader vehicles, respectively, when reaching the moment when they both reach the same speed of $V_0$. These positions are used further for calculating their respective displacement from their initial positions ($x_1^{n}$ and $x_1^{n-1}$).

In Figure 3, ${D_a}^{n,n-1}$ represents the actual distance between Vehicle *n* and $n-1$ which can be calculated using initial positions of the both vehicles according to the Equation 1:

1 $$\begin{aligned} {D_a}^{n,n-1} = x_1^{n-1} - x_1^n \end{aligned}$$

Additionally, the calculation of the minimum safety required distance between the follower vehicle and its leader can be achieved through the following equation:

2 $$\begin{aligned} {D_r}^{n,n-1} = \Delta x^n - \Delta x^{n-1} + L^{n-1} \end{aligned}$$

where ${D_r}^{n,n-1}$ represents the minimum safety required distance, $\Delta x^n$ and $\Delta x^{n-1}$ denote the respective displacements of the follower and leader vehicles until both vehicles achieve $V_0$ (calculated by $\Delta x^n$ = $x_2^n$ - $x_1^n$ and $\Delta x^{n-1}$ = $x_2^{n-1}$ - $x_1^{n-1}$), and $L^{n-1}$ is the length of the leader vehicle. In this equation, the displacement of Vehicle $n$, $\Delta x^n$, can also be determined by Equation 3, which comprises two principal components. The first component captures the distance necessary for the follower (Vehicle *n*) to react. Throughout this time interval (${t_r}^{n}$), it is assumed that the follower maintains a constant speed of $V^n$. The second component represents the distance required for the follower to adjust their speed to match that of the leader’s final speed, $V_0$, during the breaking time interval denoted as ${t_b}^{n}$ in Figure 4. In Equation 3, $a^n$ denotes the deceleration rate of the follower, which is a negative number and assumed to be constant.

3 $$\begin{aligned} \Delta x^n = V^n \cdot {t_r}^{n} + \frac{{{V_0}^2}-{{V^n}^2}}{{2a^n}} \end{aligned}$$

Similarly, the displacement of Vehicle $n-1$ can also be computed using Equation 4, which also encompasses two primary elements. The first element accounts for the distance required for the leader to attain the speed of $V_0$ during the breaking time interval denoted as ${t_b}^{n-1}$ (Figure 4), while the second element represents the distance traveled by the leader at a constant speed $V_0$, during $t_c^{n-1}$, until both vehicles reach a same constant speed, $V_0$. Here, $a^{n-1}$ denotes the deceleration rate of the leader.

4 $$\begin{aligned} \Delta x^{n-1} = \frac{{{V_0}^2}-{{V^{n-1}}^2}}{{2a^{n-1}}} +V_0 \cdot {t_c}^{n-1} \end{aligned}$$

Based on the physical relationship between variables and according to Figure 4, $t_c^{n-1}$ can be expressed by Equation 5:

5 $$\begin{aligned} t_c^{n-1} = {t_r}^{n}+{t_b}^n-{t_b}^{n-1}={t_r}^{n}+\frac{{V_0}-{V^{n}}}{{a^{n}}}-\frac{{V_0}-{V^{n-1}}}{{a^{n-1}}} \end{aligned}$$

Moreover, the reaction time of the follower can be decomposed into the driver reaction time $({t_{r_1}}^{n}> 0)$, which depends on driving behavior, and the breaking system reaction time $({t_{r_2}}^{n}> 0)$. As suggested in the literature, we can assume the breaking system reaction time equals 0.15 seconds^39^. Thus, we can reformulate the final equation as follows:

6 $$\begin{aligned} \begin{aligned} {D_r}^{n,n-1} =&(V^n \cdot ({t_{r_1}}^{n}+0.15) + \frac{{V_0^2}-{{V^n}^2}}{{2a^n}}) \\&- (\frac{{V_0^2}-{{V^{n-1}}^2}}{{2a^{n-1}}} +V_0 \cdot ({{t_{r_1}}^{n}+0.15+\frac{{V_0}-{V^{n}}}{{a^{n}}}-\frac{{V_0}-{V^{n-1}}}{{a^{n-1}}}} )) + L^{n-1} \end{aligned} \end{aligned}$$

Equation 6 calculates the minimum required safety distance between vehicles as a function of their speed, reaction time, and deceleration rate. In this equation, if we consider the leader reducing its speed to a complete stop ($V_0 = 0$), the resulting required safety distance corresponds to the classic safety distance definition in the literature. Finally, we can conclude that there is a high risk of a collision in case ${D_a}^{n,n-1} < {D_r}^{n,n-1}$.

It is critical to mention, ${D_a}^{n,n-1}$ in mountainous and low visibility roads can be considered as the visibility distance where the driver can see and understand the danger and starts breaking. Thus in places where the visibility distance ($D_v$) is lower than the required speed change distance (${D_r}^{n,n-1}$), we can consider a high risk of a crash.

**Fig. 5:**
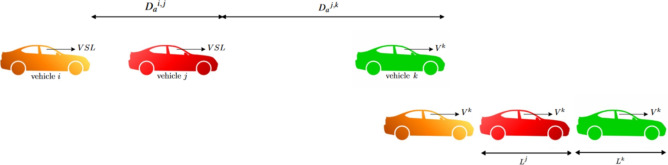
A specific one-lane car-following scenario involving three vehicles: the follower *i*, its leader *j*, and a slower-moving vehicle *k* ahead. Vehicles *i* and *j* travel at the speed imposed by the VSL sign ($V^{i}=V^{j}=V_{\textrm{SL}}$), while vehicle *k* moves at a lower constant speed $V^{k}$. The figure shows the initial spacings ${D_a}^{i,j}$ and ${D_a}^{j,k}$ as well as the vehicle lengths $L^{j}$ and $L^{k}$ used in the chain-reaction safety distance analysis (Equations 7-11).

Equation 6 can be extended to address a VSL manipulation scenario wherein a follower vehicle, denoted as Vehicle $i$, and its leader, Vehicle $j$, are both traveling at a speed indicated by the VSL sign ($V^i = V^j = VSL$). Subsequently, they initiate a sudden breaking with an equal deceleration rate, $a$, upon encountering a slower-moving vehicle, designated as Vehicle $k$, which maintains a constant speed ($V^k$) due to the congestion in front of it. Figure 5 depicts this scenario.

Here, the objective is to calculate the minimum required safety distance to prevent chain-reaction accidents. In this context, we can consider two distinct scenarios each with 2 cases, for calculating the required inter-vehicle distances:

1. Vehicle *j* does not collide with Vehicle *k*.
  1.1 Vehicle *i* does not collide with Vehicle *j*.
  1.2 Vehicle *i* collides with Vehicle *j*.
2. Vehicle *j* collides with Vehicle *k*.
  2.1 Vehicle *i* does not collide with Vehicle *j*.
  2.2 Vehicle *i* collides with Vehicle *j*.

**Fig. 6:**
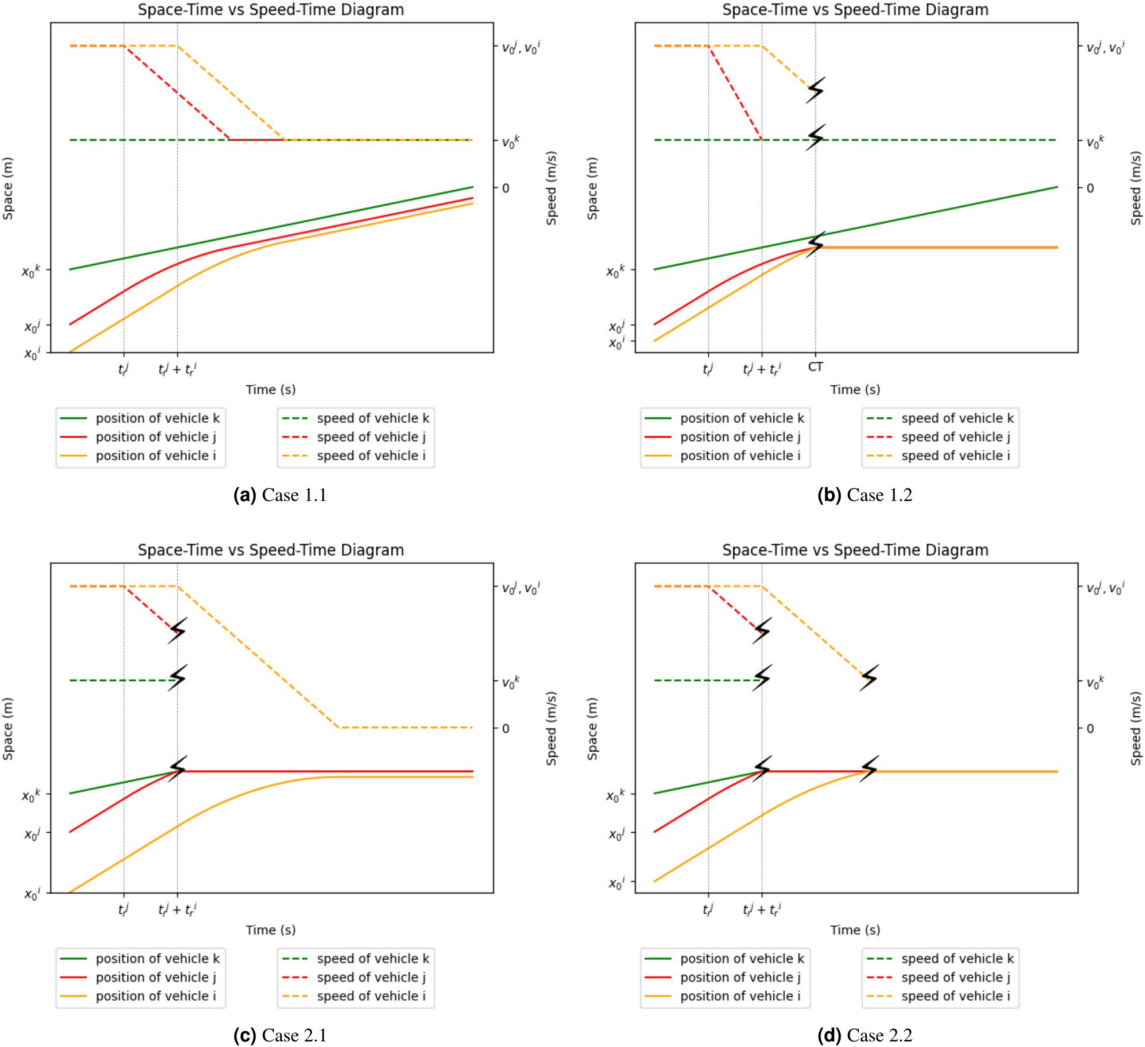
(**a** and **b**) Space-time vs speed-time diagram in Scenario 1, (**c** and **d**) Space-time vs speed-time diagram in Scenario 2.

Figure 6 illustrates the space-time vs speed-time diagrams of two possible cases within scenarios. Figure 6a represents a situation where none of Vehicles *i*, *j*, and *k* collide (Case 1.1). In contrast, Figure 6b depicts Case 1.2 where Vehicle *j* adjusts its speed to avoid colliding with Vehicle *k*, but Vehicle *i* collides with Vehicle *j*, at *CT* (collision time), due to insufficient distance for speed adaptation. For the development of both Figures (Figure 6a and Figure 6b), we assume that Vehicle *k* travels at a constant speed throughout the scenario. Vehicles *j* and *i* initially travel at constant speeds higher than that of Vehicle *k*. When deceleration begins, Vehicle *j* initiates braking immediately, while Vehicle *i* starts decelerating only after a reaction-time delay, yet both vehicles apply the same constant deceleration rate once braking begins. The space–time diagrams are computed directly from the corresponding speed–time profiles under these assumptions. The only difference between the configurations shown in Figure 6a and Figure 6b is the initial longitudinal position of Vehicle *i*. In the second case, Vehicle *i* begins closer to Vehicle *j*, resulting in a smaller initial gap and ultimately causing a collision between the two vehicles.

In these cases, the minimum required distance to avert collision between vehicles can be calculated based on Equation 6 as follows:

7 $$\begin{aligned} & {D_r}^{j,k} = VSL \cdot ({t_{r_1}}^{j} + 0.15) + \frac{{V^k}^2 - {VSL}^2}{2a} - V^k \cdot \left( {t_{r_1}}^{j} + 0.15 + \frac{V^k - VSL}{a}\right) + L^k \end{aligned}$$

8 $$\begin{aligned} & {D_r}^{i,j} = (VSL-V^k)\cdot ( {t_{r_1}}^{i}+0.15) + L^j \end{aligned}$$

We can reorganize Equation 7 to derive the form of Equation 9, which represents the required gap between Vehicle $j$ and Vehicle $k$, $G^{j,k}$, to prevent a collision. In a similar way, Equation 8 can be rearranged as 10, representing the required gap between Vehicle $i$ and Vehicle $j$, $G^{i,j}$, to prevent a collision. This reorganization expresses the result as a function of the speed difference between the two vehicles.

9 $$\begin{aligned} & G^{j,k}= {D_r}^{j,k} - L^k =( {t_{r_1}}^{j}+0.15) \cdot ( VSL - V^k) - \frac{1}{2a} \cdot {(VSL - V^k)}^2 \end{aligned}$$

10 $$\begin{aligned} & G^{i,j}={D_r}^{i,j} - L^j = (VSL-V^k)\cdot ( {t_{r_1}}^{i}+0.15) \end{aligned}$$

**Fig. 7:**
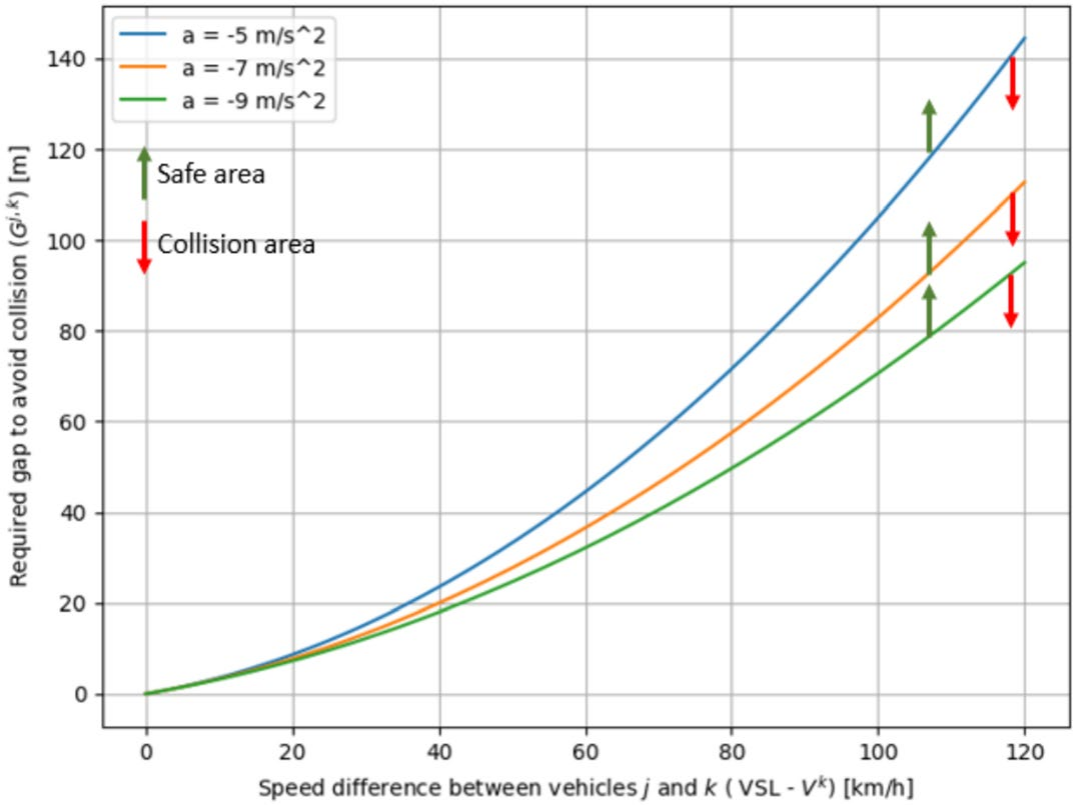
Required gap to avoid collision, $G^{j,k}$, as a function of speed difference between vehicles *j* and *k*, Equation 9, considering various deceleration rates. Gaps above the curve correspond to safe conditions, while gaps below the curve correspond to collision conditions.

Figure 7 illustrates Equation 9 ($G^{j,k}$) with $t_{r_1} = 0.85 \, \text {s}$ for various deceleration rates. This figure highlights how the required gap between vehicles can vary considerably based on the speed difference between them and the deceleration rate, which is influenced by weather and road conditions. Additionally, considering an average deceleration rate of $-7 \, \text {m/s}^2$ in emergency situations, a speed difference exceeding 50 km/h necessitates a minimum required gap of at least 25 meters. However, this requirement may not be met at certain points in the mountainous turns of our case study due to the visibility distance limit, $D_v$.

In Figure 7, the areas above the curves represent safe regions where no collisions occur if the specified gaps are maintained between vehicles. Conversely, the areas below the curves indicate crash zones. In other words, the function $G^{j,k}$ determines the boundaries of collision. In case of collision (Scenario 2), two cases can be envisaged (Case 2.1 and 2.2) as shown in Figure 6. Figure 6c presents a situation where Vehicle $i$ has enough distance to stop and avoid collision with Vehicle $j$. However, in Figure 6d, Vehicle $i$ collides with Vehicle $j$ before stopping, causing a chain accident. In such situations, the required distance to avoid a collision between vehicles $i$ and $j$, ${D_r}^{i,j}$, can be calculated by Equation 11. This is similar to Equation 7 by setting $V^{k} =0$, which is $V^j$ in this case, equal to 0.

11 $$\begin{aligned} \begin{aligned} {D_r}^{i,j} = VSL\cdot ( {t_{r_1}}^{i}+0.15) - \frac{{{VSL}^2}}{{2a}} +L^j \end{aligned} \end{aligned}$$

Figure 6c and d are constructed using the same modelling principles applied in Figure 6a and b. Specifically, Vehicles *j* and *i* begin with constant initial speeds, both higher than the lower constant speed assumed for Vehicle *k*. During the braking phase, Vehicles *j* and *i* are modelled to decelerate using a common constant deceleration rate, and the corresponding space–time trajectories are derived directly from their speed–time profiles. The distinction between the two cases illustrated in Figures 6c and 6d arises from the initial longitudinal position of Vehicle *i*. In the second configuration, Vehicle *i* starts closer to Vehicle *j*, resulting in a smaller initial gap and consequently a collision between the two vehicles. In addition, compared with the scenarios presented in Figures 6a and 6b, the initial position of Vehicle *j* relative to Vehicle *k* is reduced in Figure 6c and d. This smaller spacing similarly leads to a reduced available headway and ultimately a collision between Vehicles *j* and *k* in the lower panels.

To model the collision, it is assumed that both vehicles $j$ and $k$ come to a complete stop following the collision. Additionally, we assume that Vehicle $i$ has sufficient time to react and begins breaking. Another scenario could involve vehicles colliding during their reaction times, which implies they maintained a very short distance between them; however, this scenario is considered less probable.

**Fig. 8:**
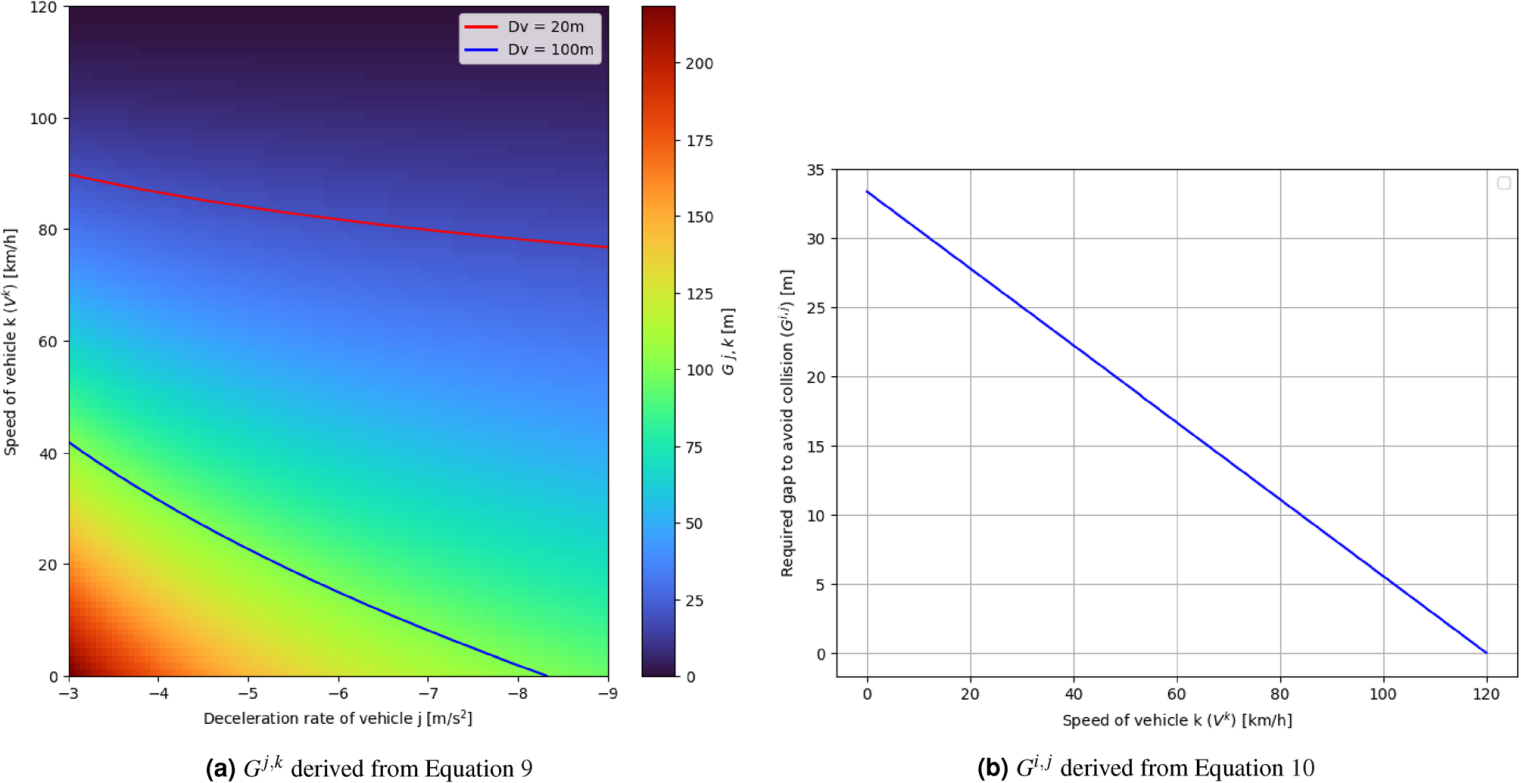
Numerical example of minimum required safe gap between vehicles in Scenario 1, VSL = 120 km/h, ${t_{r_1}}^i = {t_{r_1}}^j = 0.85$.

As an illustrative example of Scenario 1, with the VSL set at 120 km/h and identical driver reaction times for both vehicles $i$ and $j$ ($t_{r_1}^{j}=t_{r_1}^{i}=0.85 \, \text {s}$), the minimum required gap between vehicles ($G^{j,k}$ and $G^{i,j}$) may vary in relation to the speed of Vehicle $k$, $V_k$, and the deceleration rates of vehicles. This variation is visually represented in Figures 8.

Figure 8a presents the minimum required safety gap between Vehicles *j* and *k* ($G^{j,k}$) based on different deceleration rates and speed of Vehicle *k*. The visibility distance range is set between 20 and 100 meters which are denoted by $D_v$ curves in Figure 8a. These curves show the boundaries of visibility distance, which can vary based on the road and weather conditions. Here, these two values are obtained from the real test case considered for the simulation-based analysis (in the next section). As indicated in Figure 8a, the minimum required gap between vehicles to avoid a collision, $G^{j,k}$, can reach relatively high values (exceeding 100 meters) when the speed of Vehicle *k* ($V^k$) falls below 40 km/h. This speed can be considered as the congestion speed at which Vehicle *k* is driving. This underscores a heightened risk of collision if the visibility of Vehicle $j$ is limited.

Additionally, Figure 8b illustrates the minimum required gap between Vehicle $i$ and its leader, Vehicle $j$ ($G^{i,j}$), based on the speed of Vehicle *k*. Recall that both Vehicles *i* and *j* are considered to follow the VSL speed (VSL= 120 *km*/*h*). This gap varies with congestion speed ($V^k$), with a maximum value of 35 meters. Although this required gap may not be significant, it is worth mentioning that our assumption of identical driving behaviors (e.g., reaction time) and deceleration rates for both the follower and the leader, though employed for simplification, may not accurately reflect real-world conditions. As indicated in the literature^40^, heterogeneity in driving behaviors and reaction times could amplify accident risks. Following the analytical analysis in this section, we can conclude that manipulation of VSLs not only decreases safety measures but can also cause collisions and chain-reaction accidents, even on a single-lane road. Consequently, the calculated distances may escalate in diverse real-world circumstances.

**Table 1:** Notation used in the theoretical analysis section.

**Scenario**	**Symbol**	**Definition**
General scenario	*n*	Identifier of the Vehicle *n*
	$n-1$	Identifier of the Vehicle $n-1$
	$L^{n-1}$	Length of the Vehicle $n-1$
	$x_1^{n}$	Initial position of the Vehicle *n*
	$x_1^{n-1}$	Initial position of the Vehicle $n-1$
	$x_2^{n}$	Secondary position of the Vehicle *n*
	$x_2^{n-1}$	Secondary position of the Vehicle $n-1$
	$\Delta x^n$	Displacement of the Vehicle *n*
	$\Delta x^{n-1}$	Displacement of the Vehicle $n-1$
	$V^n$	Initial speed of the Vehicle *n*
	$V^{n-1}$	Initial speed of the Vehicle $n-1$
	$V_0$	Final speed of the Vehicle *n* and Vehicle $n-1$
	$a^n$	Deceleration rate of the Vehicle *n*
	$a^{n-1}$	Deceleration rate of the Vehicle $n-1$
	${t_r}^n$	Reaction time of the Vehicle *n*
	${t_{r_1}}^{n}$	Driver reaction time of the Vehicle *n*
	${t_{r_2}}^{n}$	System reaction time of the Vehicle *n*
	$t_b^{n}$	Breaking duration of the Vehicle *n*
	$t_b^{n-1}$	Breaking duration of the Vehicle $n-1$
	$t_c^{\,n-1}$	Cruising duration at of the Vehicle $n-1$ at speed $V_0$.
	$D_{a}^{n,n-1}$	Initial distance between Vehicle *n* and Vehicle $n-1$
	${D_r}^{n,n-1}$	Minimum required safety distance between Vehicle *n* and Vehicle $n-1$
	$D_{v}$	Available visibility distance
Specific one-lane car-following scenario	*i*	Identifier of the Vehicle *i*
	*j*	Identifier of the Vehicle *j*
	*k*	Identifier of the Vehicle *k*
	$L^{j}$	Length of the Vehicle *j*
	$L^{k}$	Length of the Vehicle *k*
	$V^i$	Initial speed of the Vehicle *i*
	$V^j$	Initial speed of the Vehicle *j*
	$V^k$	Speed of the Vehicle *k*
	*VSL*	Speed limit posted on VSL
	*a*	Deceleration rate
	${t_{r_1}}^{i}$	Driver reaction time the Vehicle *i*
	${t_{r_1}}^{j}$	Driver reaction time of the Vehicle *j*
	${D_a}^{i,j}$	Initial distance between Vehicle *i* and Vehicle *j*
	${D_a}^{j,k}$	Initial distance between Vehicle *j* and Vehicle *k*
	${D_r}^{i,j}$	Minimum required safety distance between Vehicle *i* and Vehicle *j*
	${D_r}^{j,k}$	Minimum required safety distance between Vehicle *j* and Vehicle *k*
	$D_v$	Available visibility distance
	$G^{i,j}$	Gap space between Vehicle *i* and Vehicle *j*
	$G^{j,k}$	Gap space between Vehicle *j* and Vehicle *k*
	*CT*	Collision time

## Problem overview and simulation results

**Fig. 9:**
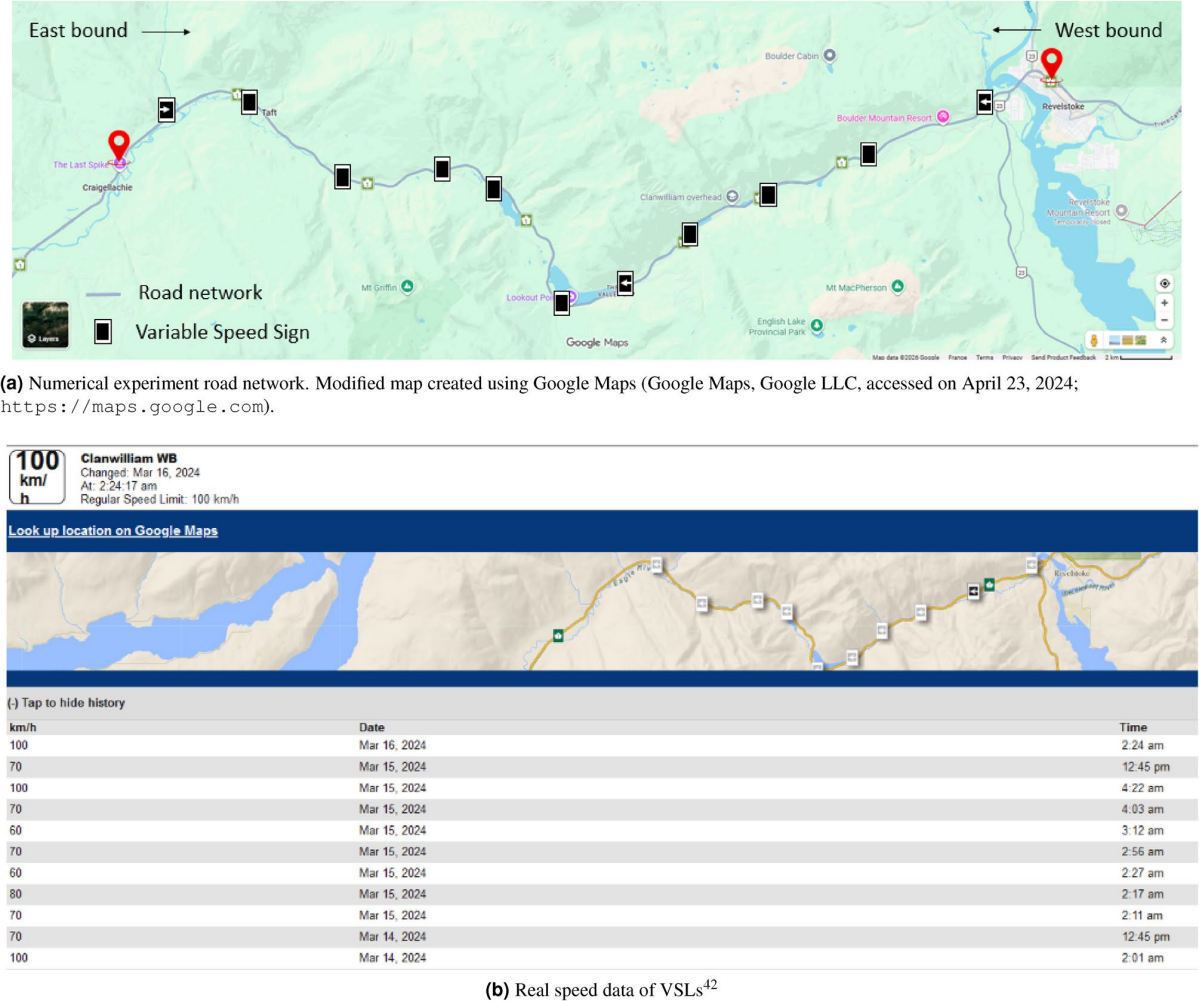
Simulation case study.

In this section, we present the implementation of different intentional disruption scenarios based on VSL manipulation on a simulated segment of Highway 1 in British Columbia, Canada, in order to examine VSL manipulation in a realistic test case. This mountainous highway is ranked among the top ten most dangerous roads in Canada in terms of the number of accidents. Specifically, we selected a 40 km stretch west of Revelstoke up to The Last Spike, as represented in Figure 9a. The simulation model developed for this study is publicly accessible on GitHub^41^.

This road network, comprising 68 nodes and 114 edges, was implemented in SUMO (Simulation of Urban MObility)^43^. The network data was obtained from OpenStreetMap, after which we corrected the speed limits, lengths, and the number of lanes based on real data collected from online maps. We implemented 10 VSLs on the westbound lane and 9 on the eastbound lane, with their actual locations and characteristics data accessible through^42^.

According to real collected data (Figure 9b), the speed limits for these variable speed limit (VSL) signs vary between 50 km/h and 100 km/h. These values are consistent with the physical roadside signage deployed along the corridor, which can also be inspected via publicly available street-level imagery services such as Google Maps. As a reference, Figure 9b presents a snapshot of real VSL data collected from^42^, wherein the speed does not exceed 100 km/h. In addition, roadside signs along the highway indicate a maximum speed limit of 100 km/h unless otherwise posted. However, such signs also imply that other traffic control devices, such as VSL systems, may recommend higher values under specific conditions. Although higher speed values than 100 km/h may be displayed by VSLs, in our numerical experiments we restrict the speed limits to a maximum of 100 km/h.

Regarding the traffic scenarios, we utilized SUMO’s default driving parameters for representing drivers’ behavior, and the traffic demand is generated using data from detectors provided on the government’s website^44^. Considering visibility is a critical factor on mountainous roads like Highway 1. We incorporated this parameter into SUMO using results from ArcGIS. We marked points on the curves of the network at 10-meter intervals and used the digital elevation data along with ArcGIS’s sight distance calculation tool to assess visibility on these curves at every 10-meter point. A linear regression was then employed to estimate visibility for intermediate points. To optimize visibility calculations in ArcGIS and reduce simulation run time in SUMO, these calculations and their integration into SUMO were limited to locations relevant to the attack scenario.

In the following subsections, we present the results of three simulated disruption scenarios, followed by a discussion. Additionally, to evaluate safety and crash risk, we utilize Safety Surrogate Measures (SSMs). These indicators are extensively employed in the literature as simulation outputs to assess the safety and the likelihood of accidents^45,46^. Specifically, we use Time To Collision (TTC) and Deceleration to Avoid a Crash (DRAC) as our principal indicators.

During the simulation, TTC and DRAC are calculated when the follower vehicle’s speed exceeds that of the leader vehicle. DRAC is determined by dividing the speed difference between the follower and the leader by the space gap between them. TTC represents the time separating a follower vehicle from its corresponding leader, calculated by dividing their space gap by their speed difference, assuming both vehicles maintain their speed and trajectory. Another SSM is “*conflict*”, which is counted when any of the mentioned measures reach a defined limit, e.g., DRAC exceeding a threshold braking value of −3 m/s² in Sumo^47^.

**Fig. 10:**
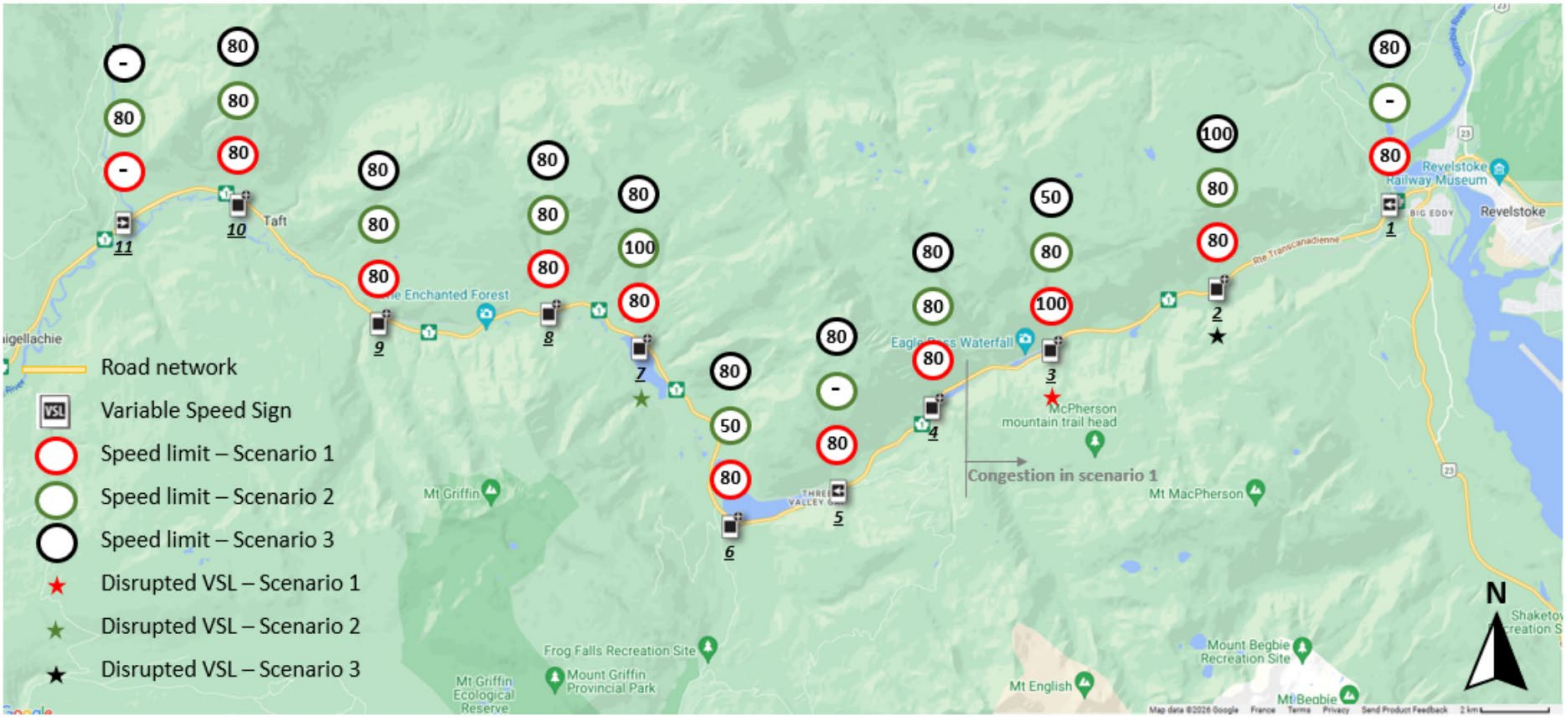
Spatial configuration of the three disruption scenarios along the study corridor. The figure shows the locations of all westbound Variable Speed Limit (VSL) signs and the corresponding posted speed limits for the attack case in each scenario. Red, green, and black circles represent the displayed speed limits in Scenario 1, Scenario 2, and Scenario 3, respectively. Similarly, red, green, and black starred symbols identify the VSLs that are disrupted in each scenario. Modified map created using Google Maps (Google Maps, Google LLC, accessed on July 1, 2024; https://maps.google.com).

The disruptions assumed to be occured between 10 AM and 4 PM. For each scenario, two cases are considered for comparison purposes. The first case represents the reference scenario in which the control center manages the speed limits. The second case represents the disruption scenario where an attacker manipulates the VSL. Details of each disruption scenario are illustrated in Figure 10. This figure shows the positions of all VSLs along the road and their respective posted speed limits in each disruption scenario. Star symbols in Fig. 10 indicate the VSLs under attack in each scenario. All non-disrupted VSLs which are not in the vicinity of the VSL under attack, are set to display a speed limit of 80 km/h as it was the median value of collected VSL data. The three scenarios considered for our examination of VSLs under attack. Figure 10 illustrates the location of the disrupted VSL. The description of these scenarios are as follows:

### Scenario 1: VSL disruption before a congestion

In this scenario, we consider traffic congestion on the westbound side of the highway, starting from upstream of VSL number 3 (Figure 10) and extending to the curve after VSL number 3, which has limited visibility. This congestion can be caused by natural events such as falling rocks, adverse weather conditions, or high traffic volume, which was observed in this test case based on^48^. Alternatively, it can be deliberately induced by an attacker. Figure 10 illustrates the details of the congestion and the location of the targeted VSL. This scenario examines two cases:

1. **Control Center Response**: Upon congestion occurrence, the control center reduces the speed limit of VSL number 3 to 50 km/h before the congestion point.
2. **Attacker Manipulation**: The attacker manipulates the VSL number 3, increasing the speed limit to 100 km/h.

**Fig. 11:**
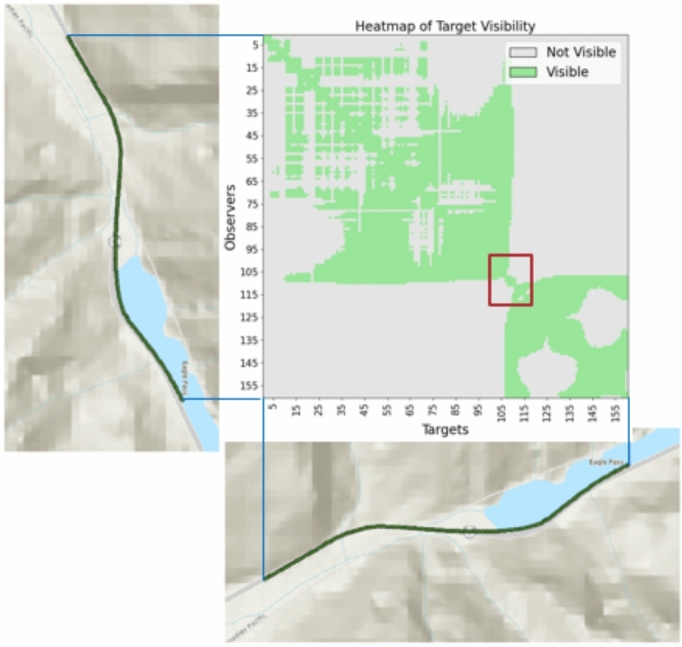
Visibility map of the curve subsequent to VSL number 3. The green points indicate the target locations along the roadway that are visible from the observer’s position on the curve, while the gray areas denote regions that are not visible due to terrain or geometric obstructions. The red box highlights the portion of the curve where visibility is limited and may lead to a higher collision risk. Modified map created using Esri ArcGIS Desktop (https://desktop.arcgis.com).

In the first case, simulation results indicate no accidents as vehicles have sufficient time to adjust their speed and stop safely behind the congestion. Conversely, in the second case, when congestion reaches the low-visibility curve (the curve after VSL number 3), vehicles crash. In the simulation, we assume each car remains stationary for five minutes after a crash, blocking the road. During the attack period, congestion queues reach the low-visibility curve twice, resulting in chain accidents involving approximately eight cars each time. Figure 11 presents the visibility map of the curve subsequent to VSL number 3. The vertical and horizontal axes represent points along the curve with 10-meter intervals. The color coding indicates whether the target point location (on the x-axis) is visible to the observer point (on the y-axis): green signifies visible status between the two points, while grey indicates non-visible status. Notably, a small segment of the curve, highlighted by a red rectangle, exhibits limited visibility. This specific region corresponds to the location where accidents occur within the simulation.

**Fig. 12:**
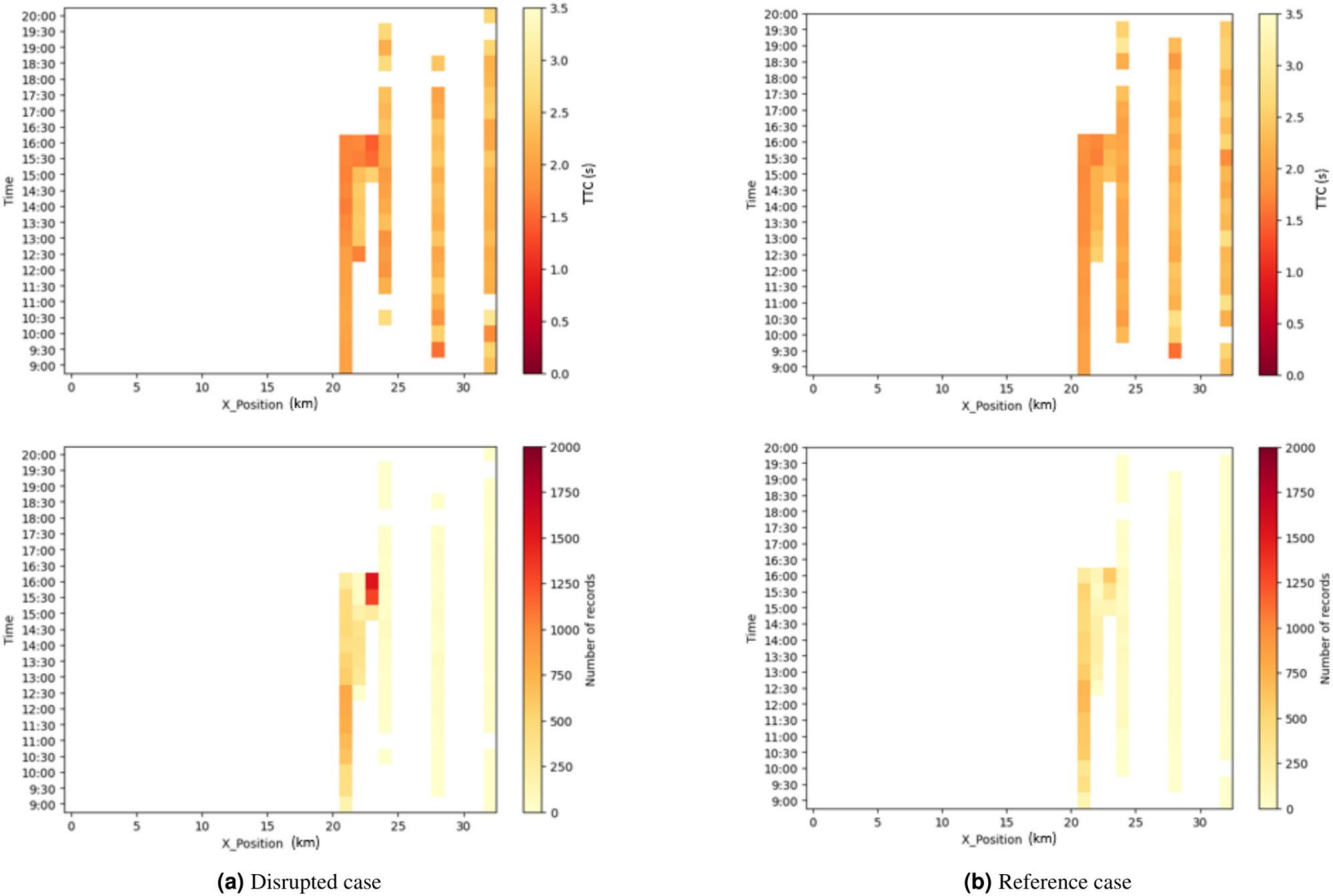
Comparison of the TTC indicator in disrupted and reference cases in Scenario 1. The figures above show the average TTC values in seconds, while the figures below display the number of conflict occurrences corresponding to these values.

Figure 12 illustrates the TTC measure in Scenario 1, comparing cases with and without disruption. The upper graphs depict the average TTC of conflicts, measured in seconds, from 9 AM to 8 PM along the westbound section of the highway. The horizontal axis represents the longitude of the network, with zero corresponding to the starting point of the road from the East. Recall that the attack duration is 6 hours (from 10 AM to 4 PM). There is a reduction in TTC after VSL number 3, between 20 and 25 km, indicating an increase in the risk of crashing.

The lower graphs display the number of conflicts corresponding to the average TTC values. These graphs reveal a significantly higher number of conflicts in the disrupted scenario, with approximately 1500 additional conflicts (about a 56% increase) compared to the non-disrupted case. This data underscores the heightened safety risks associated with the VSL under attack in this scenario. This increase predominantly occurs around 15:30 when the congestion queue reaches the low visibility part of the curve.

#### Scenario 2: speed value differences in consecutive variable speed signs

In this scenario, it is assumed that one of the VSL Signs on the eastbound side of the highway (VSL number 6) displays a speed limit of 50 km/h due to an upstream incident or adverse weather conditions. VSL number 7 is under attack in this scenario (Figure 10) As shown in Figure 7, a significant difference in speed between the VSL under attack (VSL number 7) and $V^k$, which corresponds to the subsequent VSL (VSL number 6) in this scenario, can lead to accidents or sudden breaks. Two cases are considered under this situation:

1. **Control Center Response**: The control center reduces the speed limit displayed by VSL number 7 to a reasonable limit, such as 80 km/h, to ensure a smooth transition.
2. **Attacker Manipulation**: An attacker manipulates VSL number 7 to display a speed limit of 100 km/h.

**Fig. 13:**
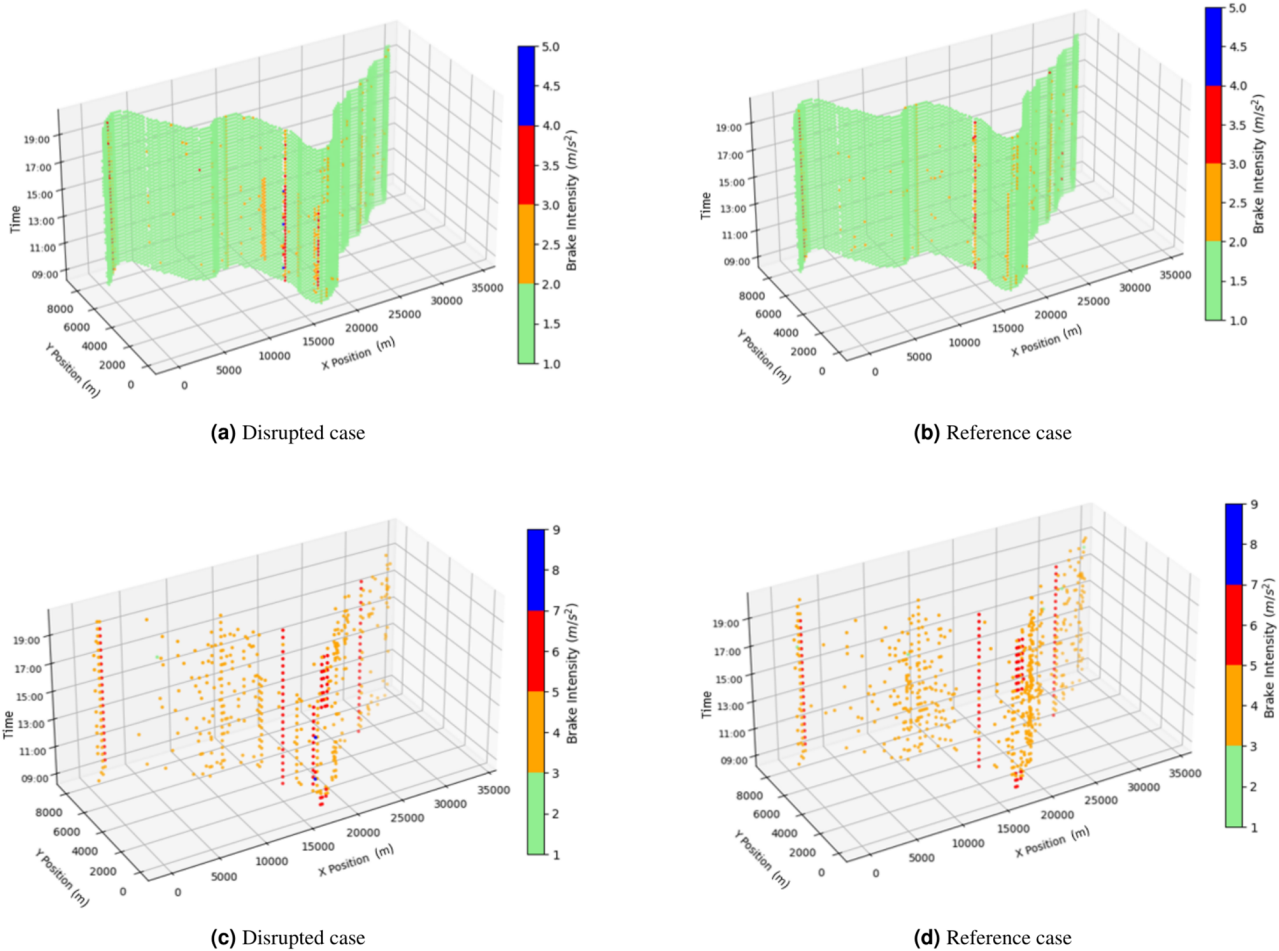
(**a** and **b**) Average braking intensity in disrupted and reference cases - Scenario 2, (**c** and **d**) Average maximum braking intensity in disrupted and reference cases - Scenario 2.

Figure 13a and b depict the absolute average of all breaking events exceeding 1 m/s$^2$ across the road from 9 AM to 8 PM. Figure 13a shows the results following the manipulation, while Figure 13b presents the results when there is no VSL disruption. Figure 13c and d compare the absolute average of maximum breaking of vehicles during their journey exceeding 1 m/s$^2$ on the network from 9 AM to 8 PM, under both disrupted and non-disrupted conditions. Note that s 13a and 13b illustrate the average breaking rate across the entire network. In contrast, Figure 13c and d represent the average of the maximum breaking rates for each vehicle within the network. Consequently, certain areas of the network appear empty in Figure 13c and d because no vehicle reaches its maximum breaking rate in those regions. As observed in both pairs of figures, there is an increased rate of hard breaking in the disrupted scenario. In both pairs of figures, we observe the presence of average breaking intensities exceeding −4 $m/s^2$ in disrupted scenarios. While in the disrupted case (Figure 13b), 31% increase is reported in the number of breaking events more severe than −4 m/s² in the disrupted case. These hard-breaking events predominantly occur at road segments where there is a lane merge or a speed transition from 100 km/h to 50 km/h. Although no accidents are reported in this scenario, the elevated hard-breaking rates pose potential safety issues. The effect of speed difference on safety has also been studied through the analysis of crash data by^49^, providing evidence of a higher likelihood of injury or fatality associated with greater speed differences in freeway rear-end crashes.

#### Scenario 3: VSL manipulation near lane merging points

This scenario is similar to Scenario 2, but on the westbound side of the high way and a disruption on VSL number 2 with a critical distinction: the VSL Sign displaying a speed limit of 50 km/h (VSL number 3) is positioned directly after a location wherein tow lanes are merged result in road capacity reduction. Figure 10 illustrates the location of the targeted VSL, with the adverse weather or incident occurring after VSL number 3, which is set to display 50 km/h. The two cases in this scenario are:

1. **Control Center Response**: The control center reduces the speed limit displayed by VSL number 2 to a reasonable limit, such as 80 km/h, to ensure a smooth transition.
2. **Attacker Manipulation**: An attacker manipulates VSL number 2 to display a speed limit of 100 km/h.

In this scenario, as the speed change is located in a critical area, where visibility is limited and there is a merging from two lane to one lane, multiple chain accidents happens. Recall that crashed cars in the simulation are set to stop in the middle of the way for 5 minutes, modeling the time that it takes to move the cars into the corridor/road shoulders.

In the reference situation, the control center adjusts the VSL to reflect real-time conditions, reducing the speed limit to 80 km/h, resulting in no accidents. In contrast, in the scenario under attack, the initial accident occurs around 13:00 when a group of vehicles traveling at 100 km/h attempts to slow down to 50 km/h while merging. Within the first five minutes after the initial crash, a chain reaction resulted in 12 cars colliding due to poor visibility and significant speed differences.

**Fig. 14:**
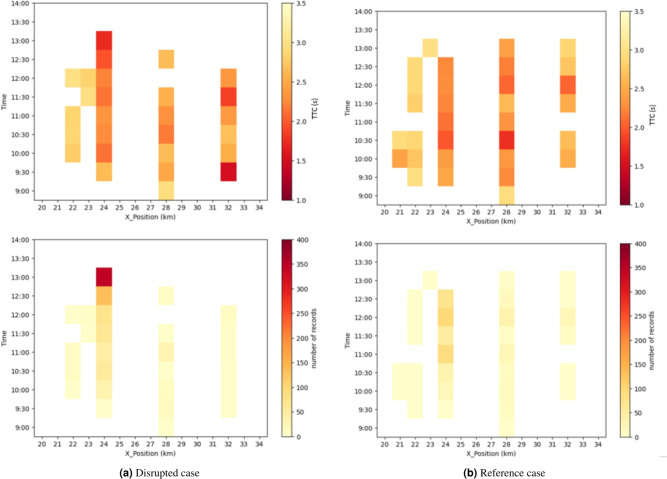
Comparison of the TTC indicator in disrupted and reference cases in Scenario 3. The figures above show the average TTC values in seconds, while the figures below display the number of conflict occurrences corresponding to these values.

Figure 14 presents the TTC measure of conflict situations over 15 kilometers of highway between VSL number 3 and VSL number 2 from 9 AM to five minutes after the first accident. The upper graphs show the average TTC of conflicts, measured in seconds. In the disrupted scenario, there is a reduction in TTC after VSL number 2 over 24 kilometers of the network, indicating an increased risk of crashes. The lower graphs display the number of conflicts corresponding to the average TTC values. These graphs reveal a significantly higher number of conflicts in the disrupted scenario, with approximately 325 additional conflicts compared to the reference case. This data underscores the heightened safety risks associated with the VSL under attack in this scenario. The increase predominantly occurs around hour 13:00, when the first accident happens. There are some additional conflicts at km 28 in the reference scenario, but they are negligible due to their low frequency and may result from simulator parameters, e.g., the start lane or initial vehicle speed. This analysis is limited to the first five minutes after the first accident, reflecting real-world conditions where immediate actions are typically taken to prevent further chain accidents, such as placing warning signs hundreds of meters ahead to alert arriving drivers.

**Fig. 15:**
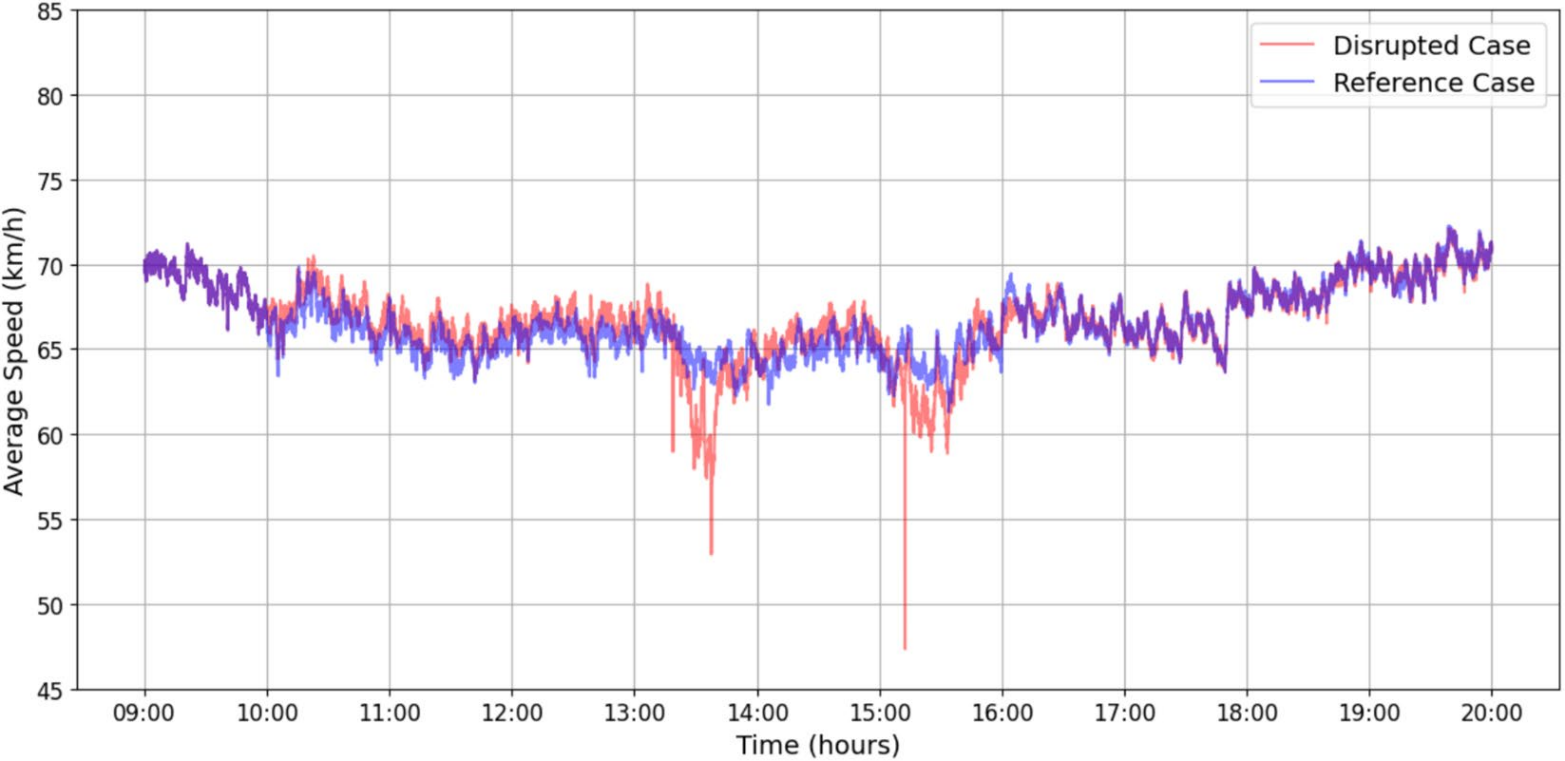
Average network speed in disrupted and reference cases of Scenario 3 from 9 AM to 8 PM.

Figure 15 depicts the average network speed from 9:00 AM to 8:00 PM in the extreme scenario where no interventions occur after the initial five minutes. In this case, due to the extended stretch of low visibility along the curve between VSL number 2 and VSL number 3, numerous chain accidents occur consecutively. The results demonstrate considerable reductions in average network speed during accidents, dropping from approximately 65 km/h to 55 km/h or lower. This decrease can have negative impacts on network performance and travel costs.

All the scenarios above highlight the potential impacts and dangers of VSL manipulation on traffic flow and driver safety. In the subsequent section, we discuss the role of key variables in the effectiveness of VSL attacks and propose possible mitigation solutions.

## Discussion

As presented, the results indicate that disruptions can vary in their effectiveness in terms of generated cost, time loss, and accident frequency. The total cost may increase if the attacker strategically selects the optimal time and location for the disruption^21^. In the following, we discuss the impact of these two key variables on the effectiveness of the attack followed by an analysis of how diverse driver behaviors affect traffic dynamics.

**Fig. 16:**
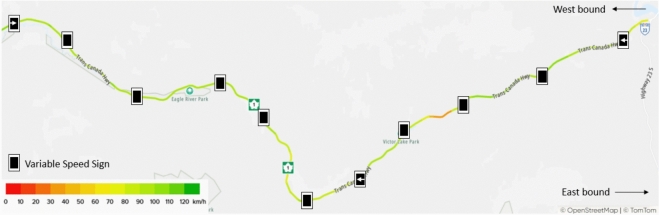
Average speed of the network from 10 AM to 4 PM. Modified map created using TomTom website^50^, data of 10/04/2022.

In all scenarios, attacks occur during peak hours when the network experiences high traffic flow. Additionally, in Scenario 1, we simulate congestion within the network, suggesting that an attacker might either exploit natural congestion or deliberately induce it through a coordinated attack. Figure 16 shows the average speed of the network for a specific day from 10 AM to 4 PM, as reported by the TomTom MOVE web portal^50^. The figure shows a speed reduction after VSL number 3 (which is assumed in scenarios 1 and 3). This historical data is readily accessible online, providing attackers with valuable insights into traffic conditions. Moreover, real-time speed information available on platforms like Google Maps further enhances the potential for identifying the critical time and location for an effective attack.

**Fig. 17:**
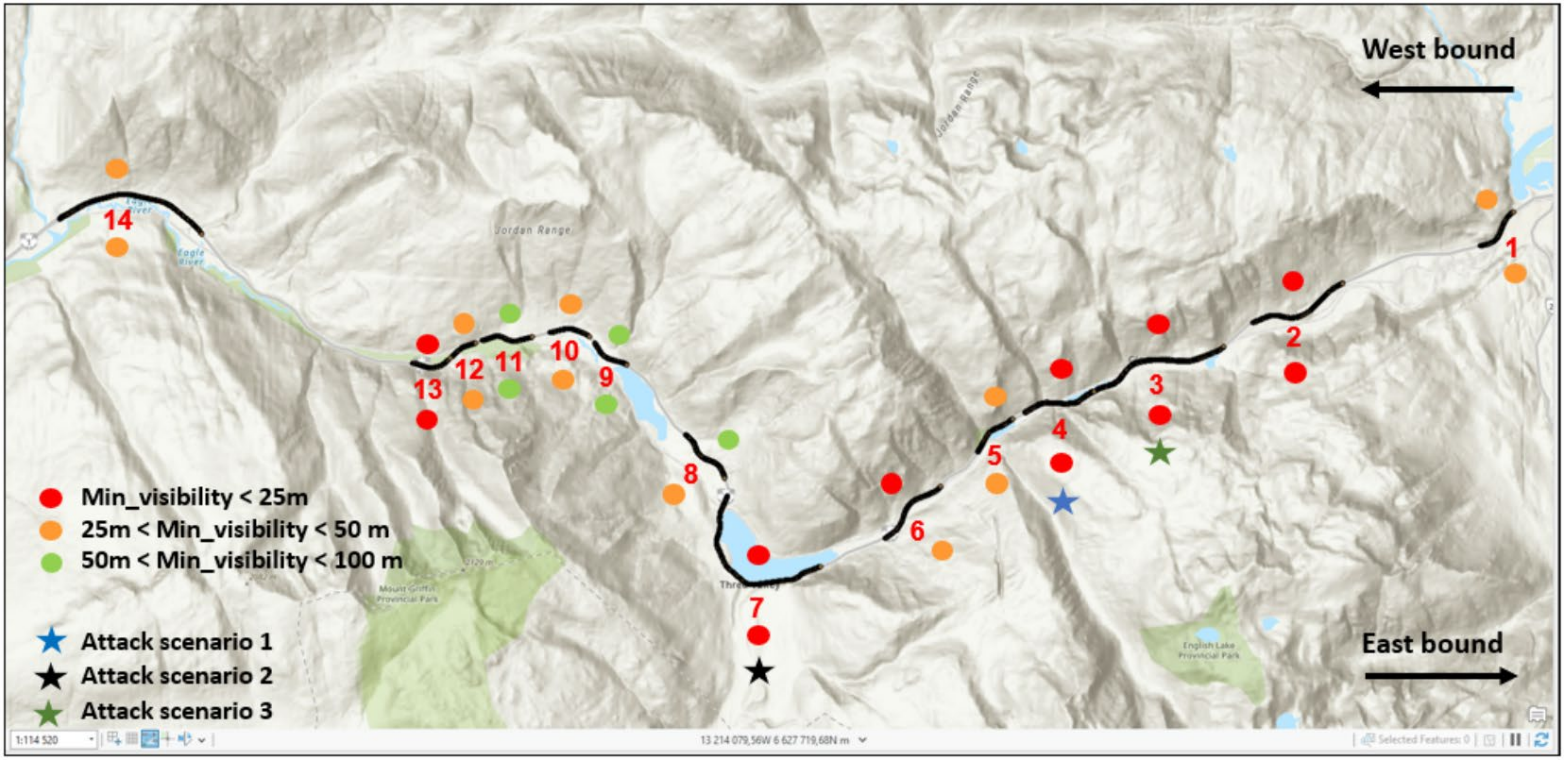
Visibility distance along the test corridor. Colored circles represent the minimum visibility distance at each curve: red (< 25 m), orange (25–50 m), and green (50–100 m). Visibility values shown above the roadway correspond to the westbound direction, while those below the roadway correspond to the eastbound direction. Starred symbols mark the VSLs manipulated in the three attack scenarios. Modified map created using Esri ArcGIS Desktop (https://desktop.arcgis.com).

Figure 17 illustrates the visibility measure on various curves within the network shape. The visibility of westbound curves is indicated above the road, while the visibility of eastbound curves is presented below the road. All the scenarios examined involve curves with low visibility, characterized by segments where the visibility distance is less than 25 meters. In Scenario 1, as congestion spreads and reaches these low-visibility segments, a chain reaction of accidents is observed. Conversely, in Scenario 2, although there are points with low visibility between VSLs number 6 and 7, the attack does not result in accidents as the location affected by the attack is not among low-visibility areas. In Scenario 3, accidents arise not only due to visibility constraints but also because of the location of merging lanes. In this scenario, the targeted VSL is positioned about 70 meters before the point where the road narrows from two lanes to one. This setup causes sudden breaking at merging location, leading to accidents.

Another critical parameter that influences various driving decisions, including speed, spacing, acceleration, and deceleration, is driver behavior. this parameter collectively shape traffic dynamics and road user safety. In this study, the default driver behavior in SUMO was applied in all scenarios. To incorporate diverse driver behaviors into the simulation, detailed traffic data specific to the case study is required; however, such data is unavailable for the present analysis.

To have a more realistic scenario and assess the extent to which variations in driver behavior influence simulation results, we tested the first scenario using three distinct driver profiles: *cautious*, *normal*, and *aggressive* as it has been demonstrated that speed choice of drivers is influenced by a wide range of personal factors^51^. Each profile was characterized by a unique desired speed distribution, modeled using the speedFactor parameter in SUMO. This parameter acts as a multiplier for the speed limit, enabling variability in driver behavior by allowing speeds to exceed or fall below the designated limit. The speedFactor parameter is defined by a normal distribution, as implemented using the normc function. The formulations of the SUMO’s speed factor is provided in Equation 12:

12 $$\begin{aligned} \text {speedFactor} = \text {normc}(\text {mean}, \text {standard deviation}, \text {lower bound}, \text {upper bound}) \end{aligned}$$

Here (Equation 12), the first parameter represents the mean value of speed factor, while the second is the standard deviation. The remaining two parameters define the lower and upper bounds for the distribution.

Table 2 outlines the parameters of the speed factor assumed for each driving behavior. The normal profile uses the default speed factor of SUMO, whereas the cautious and aggressive profiles incorporate a deviation of 20% from the normal case.

**Table 2:** Speed Factor parameters for different behaviors.

**Behavior**	**Mean**	**Std. Dev.**	**Lower Bound**	**Upper Bound**
Aggressive	1.2	0.1	0.2	2
Normal	1.0	0.1	0.2	2
Cautious	0.8	0.1	0.2	2

**Fig. 18:**
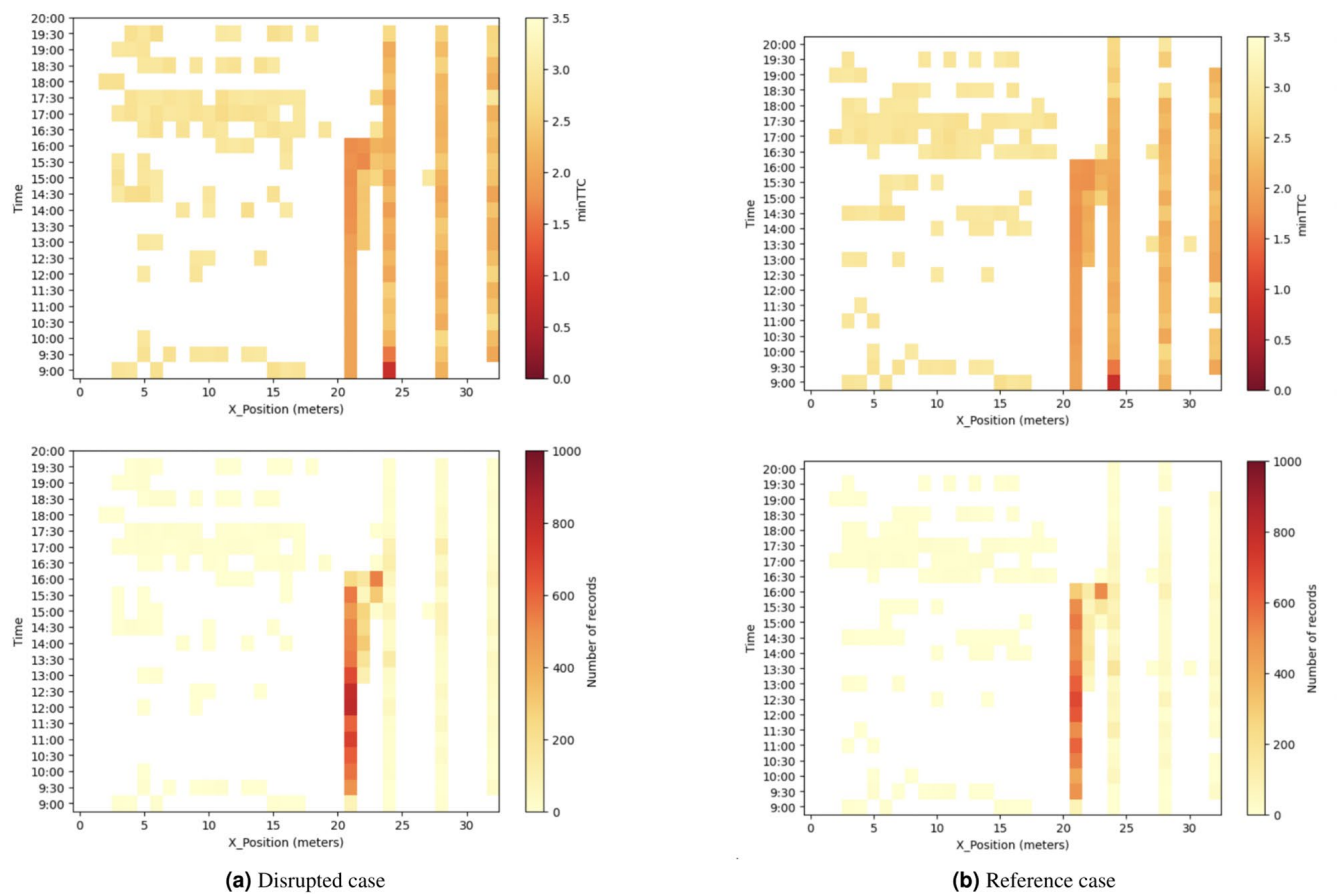
Comparison of the TTC indicator in disrupted and reference cases in Scenario 1 with different driver behaviors. The figures above show the average TTC values in seconds, while the figures below display the number of conflict occurrences corresponding to these values.

Figure 18 illustrates the results of Scenario 1, considering a distribution of driver categories as follows: 40% cautious drivers, 40% normal drivers, and 20% aggressive drivers. This distribution is adopted based on^52^, as the real data for our case study was not available. As shown, the total number of conflicts decreases in both the attack and mitigation cases compared to the baseline scenario (Scenario 1, Figure 12), where all drivers are classified as normal.

This reduction, particularly near the low-visibility area, can be attributed to the high proportion of cautious drivers, 40%, included in the simulation. However, despite this overall decrease, an attack on VSL still introduces safety risks and increases the chance of accidents. Moreover, a noticeable increase in the dispersion of conflicts across the entire highway is observed during the simulation period, compared to the baseline scenario (Figure 12). This phenomenon likely stems from the heterogeneity in driver behavior introduced in this scenario, contrasting with the uniform driver behavior in Scenario 1, where all drivers were classified as normal.

Furthermore, we used the default parameters of SUMO, where the emergency acceleration is set to −9 m/s². However, this parameter can be adjusted according to specific weather and road conditions, potentially altering the outcomes.

To quantify variability arising from stochastic elements in the simulation environment, we conducted more than 100 independent Monte-Carlo runs for the first scenario. Each run was initialized with a unique random seed, while maintaining the same demand and driver-type distribution (20% aggressive, 40% normal, 40% cautious). For each run, we measured the number of collisions and the percentage increase in conflict events between the attack and reference conditions.

The statistics show that, on average, the attack leads to a 24.95% increase in conflicts, with a standard deviation of 12.46%, indicating moderate variability across seeds. The increase of 21.88%–28.01% conflicts compared with the baseline, with a 95% confidence interval, suggests that the reduction in safety level remains significant even under the most conservative estimation. Similarly, the number of collisions confirms a consistent rise in collision occurrences across the repeated trials. These results demonstrate that the attack effect is robust and not an artifact of a specific random seed. The detailed statistics are summarized in Table 3.

**Table 3:** Summary of uncertainty analysis over more than 100 independent simulation runs.

**Metric**	**Mean**	**Std. Dev.**	**95% CI**
Increase in Conflicts (%)	24.95%	12.46%	21.88% – 28.01%
Number of Collisions	21.82	12.87	18.66–24.98

This study primarily focuses on identifying and exploring the cyber-physical security vulnerabilities in VSLs. However, it is essential to briefly address potential solutions. The vulnerabilities discussed can be mitigated through multiple layers of protection within both the cyber and physical systems.

In the cyber domain, employing secure communication protocols, such as SNMPv3 with authentication and encryption, alongside frequent software updates, can significantly reduce the likelihood of attackers gaining access to the network, thereby enhancing overall security^36^. Adopting a zero-trust framework in ITS can help address the challenges posed by stolen credentials and insider threats. However, such changes may not only require hardware upgrades but also a shift in cyber hygiene culture and standard operating procedures, necessitating training and ongoing education for staff.

It is crucial to recognize that solutions for cyber-physical systems, such as VSLs in transportation, should not be confined solely to information technology (IT)-based approaches. Physical system-based solutions must also be considered as a last line of defense when cyber-based security measures fail, particularly in the case of zero-day or zero-knowledge attacks. Operational Technology (OT) data can be leveraged to create intrusion detection systems that use patterns from diverse data sources, such as historical speed and weather data, to identify anomalies and distinguish between attack data and legitimate commands. In these systems, real-time data and speed limits are compared to historical patterns. For instance, linking VSLs with upstream flow or speed detectors can help align speed limits with actual traffic conditions, preventing high-speed settings in areas of congestion. Additionally, incorporating real-time speed data collected from probe vehicles or GPS systems can allow for the adjustment of speed limits based on current traffic conditions, minimizing discrepancies between posted speed limits and actual vehicle speeds. This study also attempted to identify critical locations for the deployment of VSLs. A complementary mitigation strategy is to avoid installing these systems in high-risk areas, such as merging points.

Another potential mitigation strategy involves introducing restrictions on VSLs. Implementing minimum or maximum speed limits, along with limiting the rate of speed changes, can complicate attacks. These measures may hinder attackers while avoiding the need for additional communication channels, thereby reducing the potential attack surface. However, such limitations may also negatively impact network performance or safety in scenarios without an attack. For example, setting a minimum speed limit or restricting the rate of speed change could prevent the reduction of speed to safe levels during emergencies, such as a rockfall. Therefore, these limitations must be carefully assessed before implementation.

Rather than independently and permanently limiting the speed on each VSL, dynamic rules based on external data can be used to evaluate changes in speed limits. For example, linking consecutive VSLs can prevent significant discrepancies between their speed limits by comparing values across the network. As an illustrative example, we applied rule-based constraints to the final scenario. Specifically, the control center or an attacker is restricted from increasing the speed limit of consecutive VSLs by more than 20 km/h. In Scenario 3, this mitigation rule means that VSL 2, the target of the attack, cannot exceed 70 km/h when VSL 3 is set to 50 km/h.

**Fig. 19:**
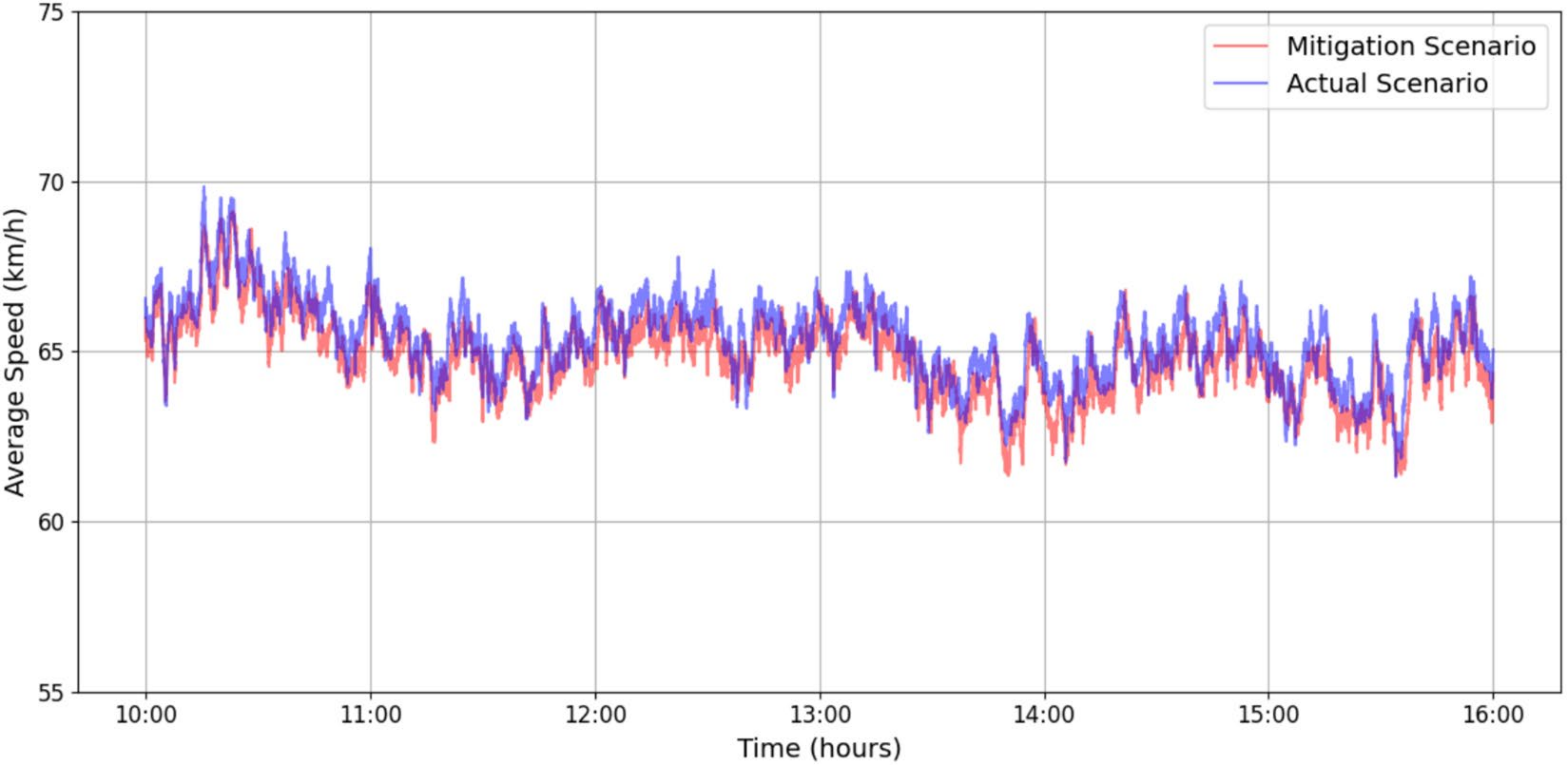
Comparison of the average network speed (from 10 AM to 4 PM) with and without the mitigation strategy.

While this mitigation strategy can prevent an attack from negatively impacting traffic conditions or safety, it may also impair network performance by limiting the maximum speed in normal conditions. Figure 19 compares the average network speed with and without the speed difference constraint from 10 AM to 4 PM. The purple line represents the average network speed when the control center sets the speed limit to 80 km/h, while the pink line reflects the same scenario with the mitigation applied, limiting the speed to 70 km/h for VSL 2. The results show a slight decrease in average network speed when VSL 2 is set to 70 km/h, with a difference of approximately 1.7 km/h. This discrepancy may become more significant in scenarios where there are larger differences in speed limits between the mitigated and non-mitigated conditions. Therefore, it is essential to thoroughly test proposed mitigation strategies before their implementation.

This discussion emphasizes not the prioritization of one solution over another but rather the complex, multi-faceted nature of cyber-physical systems. It highlights the necessity of employing multiple layers of defense against cyberattacks. Finding appropriate, case-specific solutions requires detailed analysis and the development of tailored strategies.

## Conclusion

This paper explored the impact of intentional disruptions on Variable Speed Limit (VSL) singes and outlined a cyber-physical framework for VSL systems, identifying potential attack points. While previous literature has explored the effects of VSLs on congestion, safety, and environmental factors, and has suggested various algorithms to enhance these aspects, it has not thoroughly examined VSL vulnerabilities and their impacts on traffic safety under cyberattacks. This study reveals that manipulating roadside controllers is more straightforward than tampering with sensors or intercepting wireless communications, assuming that encryption and authentication are effectively implemented. We also formulated an analytical model to evaluate accident risks during car-following scenarios under attack, focusing on the formulation of the required distance between vehicles to avoid collision based on speed differences and deceleration rates. Then, a real test case of Highway 1 in British Columbia, Canada, was considered for simulation-based analysis of VSL under attack. Our findings indicate that VSLs, which are primarily implemented to enhance safety on dangerous roads, may paradoxically introduce safety risks if not adequately secured and monitored. Simulations of various disruption scenarios demonstrated that three following factors can lead to sudden braking and/or accidents, especially on mountainous roads with limited visibility: (i) substantial speed variations between adjacent VSLs (Fig. 7); (ii) poor placement of VSLs; (iii) significant discrepancies between posted and actual traffic speeds. It is important to note that, in all scenarios considered, the attack targeted a single VSL to illustrate that even a localized disruption can significantly affect traffic flow and safety with minimal effort. However, this analysis can be extended to scenarios that involve multiple VSLs, which could potentially increase disruptions and exacerbate safety risks. We also proposed several mitigation strategies and tested one of them; however, assessing the effectiveness of these strategies remains an area for future research, possibly through field experiments. Another limitation of this study is that detailed traffic data was not utilized. Because the primary objective was to assess the safety risks associated with VSL manipulation rather than to perform a fully calibrated traffic simulation, we relied on publicly available detector flow data to generate network demand. Nevertheless, the simulation results could be further improved through calibration of driver behaviour if more detailed traffic data becomes available. Additionally, our case study was conducted on a predominantly single-lane mountainous highway; extending this work to multi-lane networks with complex vehicle interactions would be a valuable direction for future studies.

## Data Availability

The datasets supporting the findings of this study are available as follows: - The actual locations and characteristics of the Variable Speed Limit (VSL) signs are publicly accessible via the DriveBC repository: https://vsls.drivebc.ca/. - The traffic demand data used in the simulations were generated using data from detectors available on the British Columbia government’s website: https://twm.th.gov.bc.ca/?c=tdp&lon=-123&lat=54.5&z=5&sb=1 &. - Visibility assessments were conducted using ArcGIS using digital elevation data, accessible via https://www.arcgis.com/, combined with ArcGIS’s sight distance calculation tool to evaluate visibility at each location on Highway 1. All the relevant code and simulation files have been made available for academic use. The repository can be accessed at the following link: https://github.com/MarMar-s/Cybersecurity-Posture-of-Variable-Speed-Limit-Signs-Revelstoke-Case-Study.git
